# New insights on human IRE1 tetramer structures based on molecular modeling

**DOI:** 10.1038/s41598-020-74347-8

**Published:** 2020-10-15

**Authors:** Antonio Carlesso, Johanna Hörberg, Anna Reymer, Leif A. Eriksson

**Affiliations:** 1 Department of Chemistry and Molecular Biology, University of Gothenburg, 405 30 Göteborg, Sweden; 2 Faculty of Biomedical Sciences, Institute of Computational Science, Università della Svizzera italiana (USI), Lugano, Switzerland

**Keywords:** Computational chemistry, Molecular dynamics, Structure prediction, Proteins

## Abstract

Inositol-Requiring Enzyme 1α (IRE1α; hereafter IRE1) is a transmembrane kinase/ribonuclease protein related with the unfolded protein response (UPR) signaling. Experimental evidence suggests that IRE1 forms several three dimensional (3D) structural variants: dimers, tetramers and higher order oligomers, where each structural variant can contain different IRE1 conformers in different arrangements. For example, studies have shown that two sets of IRE1 dimers exist; a face-to-face dimer and a back-to-back dimer, with the latter considered the important unit for UPR signaling propagation. However, the structural configuration and mechanistic details of the biologically important IRE1 tetramers are limited. Here, we combine protein–protein docking with molecular dynamics simulations to derive human IRE1 tetramer models and identify a molecular mechanism of IRE1 activation. To validate the derived models of the human IRE1 tetramer, we compare the dynamic behavior of the models with the yeast IRE1 tetramer crystallographic structure. We show that IRE1 tetramer conformational changes could be linked to the initiation of the unconventional splicing of mRNA encoding X-box binding protein-1 (XBP1), which allows for the expression of the transcription factor XBP1s (XBP1 spliced). The derived IRE1 tetrameric models bring new mechanistic insights about the IRE1 molecular activation mechanism by describing the IRE1 tetramers as active protagonists accommodating the XBP1 substrate.

## Introduction

The unfolded protein response (UPR) is a conserved set of signaling pathways in the endoplasmic reticulum (ER) that arise from an imbalance between the ER machinery and the accumulation of misfolded proteins^1^. The accumulation of misfolded proteins drives the activation of three transmembrane proteins: Inositol-Requiring enzyme 1α (IRE1), protein kinase R (PKR)-like ER kinase (PERK), and activating transcription factor 6 (ATF6)^1^. These proteins trigger a series of cellular responses including upregulation of UPR target genes, translation attenuation, and ER-associated degradation (ERAD)^2^ with the aim to push the cell towards either survival or apoptosis.

**Figure 1:**
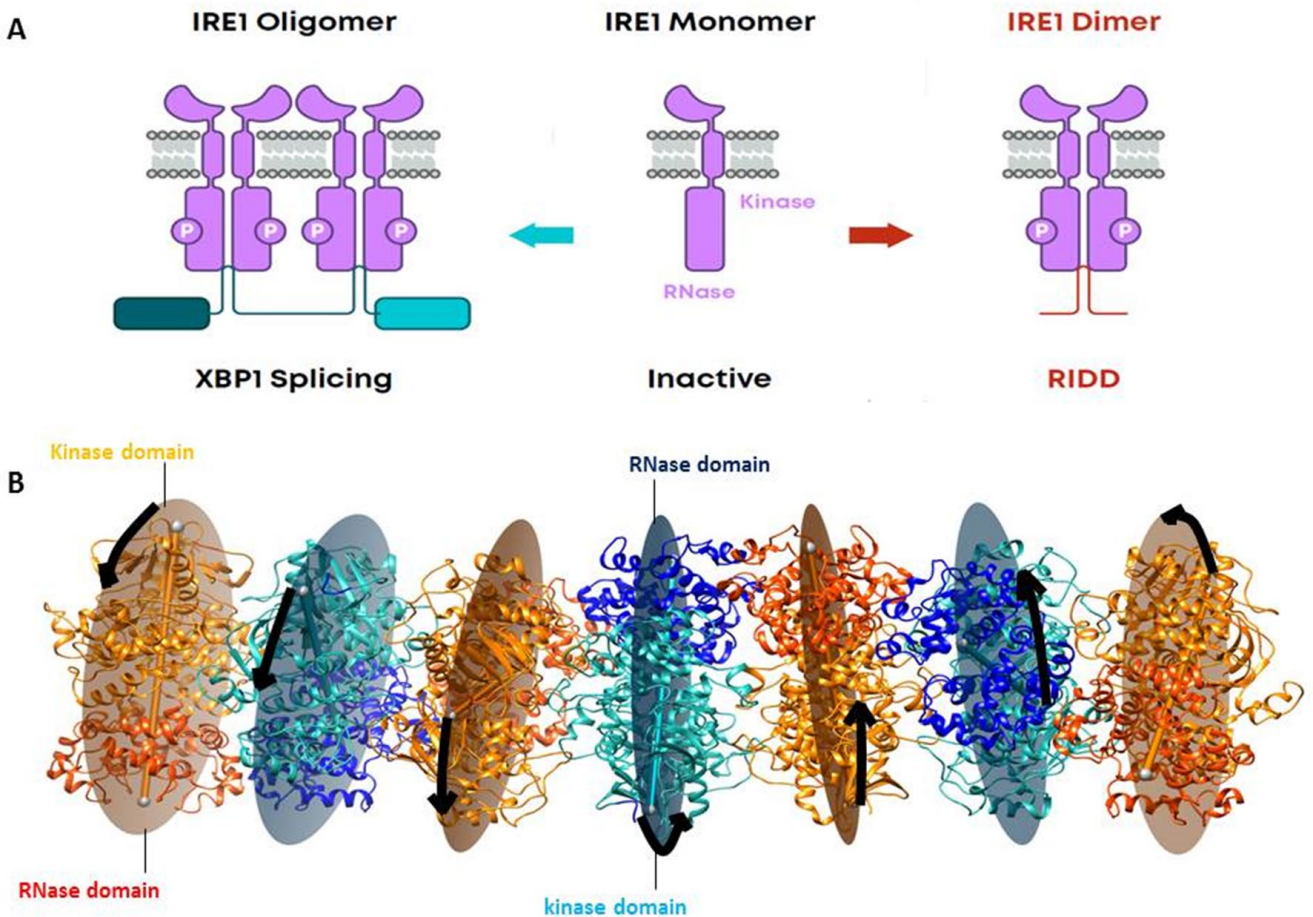
(**A**) General scheme of IRE1 activation. (**B**) Sideways view of yeast IRE1 oligomeric configuration (PDB code: 3FBV^5^); from one dimer pair to the next, the system displays a 52° right hand rotation as shown from the dimer axes passing through centers of mass (COM) at the dimer interfaces within dimer plane schematic representation. Each dimer pair is in back-to-back conformation, and the dimers are shown as alternating orange (kinase domain)/red (RNase domain), and light green (kinase domain)/blue (RNase domain), respectively. Protein images produced using UCSF Chimera 1.14, https://www.cgl.ucsf.edu/chimera.

IRE1 represents the most evolutionary conserved branch of the UPR signaling pathway. The protein is structurally organized into three domains: an N-terminal luminal domain, a transmembrane domain and a cytosolic domain. The cytosolic domain, in turn, is composed of two catalytic domains: a kinase and an RNase domain^2^. Several experimental studies such as X-ray crystallography^3^, live cell microscopy^4^, kinetic studies of RNA cleavage^5^, Western blots, microscopy and image analysis^6^, and in vitro cleavage and splicing assays^7^ have provide mechanistic insights on IRE1 activation^3,6,8^: IRE1 forms dimers, tetramers, and larger order oligomers. Upon IRE1 activation, the protein dimerizes into a face-to-face dimer (Supplementary Fig. S1) to allow trans-autophosphorylation of the kinase domains. Following trans-autophosphorylation, IRE1 reorganizes into a back-to-back dimer (Supplementary Fig. S1) or higher order oligomers of dimers to activate the RNase domain^2,6^. The activated RNase domain excises a 26-nucleotide intron from the X-Box Binding Protein 1 (XBP1) mRNA^2^, resulting in the active transcription factor that drives the expression of UPR target genes to push the cell towards the survival state^2^. Removal of the intron occurs through splicing of two stem-loops^7^ where one IRE1 dimer is required for each single cleavage event^5,7,9^. This suggests that at least an IRE1 tetramer is required to complete the splicing reaction of XBP1 (Fig. 1A). Continuous accumulation of misfolded proteins leads to high ER-stress conditions, which increases the IRE1 splicing of other ER-bound RNA in a process known as regulated IRE1-dependent decay (RIDD; Fig. 1A)^10,11^. The RIDD-pathway pushes the cell towards apoptosis.

The structures of the two distinct IRE1 dimer conformers (i.e. face-to-face and back-to-back) and higher oligomers have been solved^3,8^; the oligomeric kinase-ribonuclease cytosolic domain of the yeast IRE1 crystallographic structure (PDB code: 3FBV) is shown in Fig. 1B; in Supplementary Fig. S1 a cartoon representation is added, to further illustrate the 52° rotation from one dimer to the next. The structural recurrence of the face-to-face and back-to-back dimer structures from yeast to human cells suggests their importance in UPR signaling^3^. Although the splicing of XBP1 implies an important role of IRE1 oligomers, the mechanistic details are limited. The two available crystal structures of yeast IRE1 oligomers where each dimer pair shows a back-to-back conformation (PDB code 3FBV and 3SDM with resolution 3.3 and 6.6 Å, respectively)^3^ provide a limited, static picture, and also raises questions as it suggests an unrealistic curvature of the ER membrane. To this end, the determination of a high-resolution structure of the human IRE1 tetramer and knowledge of its conformational dynamics is central for a more complete understanding of IRE1 activation and ER-related RNA splicing in human cells.

In the present study, we combine protein–protein docking studies^12^ with extensive Molecular Dynamics simulations in the microsecond range to derive an all-atom model of the human IRE1 tetramer and investigate the molecular mechanism of IRE1 activation. Firstly, we verify the ability of the protein–protein docking protocol to reproduce the IRE1 back-to-back crystallographic complex. Secondly, we investigate several possible conformations of human IRE1 tetramers, obtained by docking from the IRE1 back-to-back dimer. Finally, we subject our derived IRE1 tetramer models to Molecular Dynamics (MD) simulations^13^. Through analyses of the trajectories, we collect new structural insights on the molecular conformations and mechanisms that lead to IRE1 activation and XBP1 splicing.

## Methods

### Selection and preparation of IRE1 crystal structures

The Schrödinger protein preparation wizard tool^14^ was used to prepare the IRE1 crystal structures: the staurosporine bound human back-to-back dimer (PDB ID: 4YZC^15^) and the yeast oligomeric structure (PDB ID: 3FBV^5^). The structure of the yeast IRE1 tetramer was obtained from the oligomeric structure by deleting chains E-N and excluding co-crystallized ligands in the kinase pockets. Missing loops were generated using Prime^16^ and hydrogen atoms were added. The protonation and tautomeric states of Asp, Glu, Arg, Lys and His were determined to match a pH of 7.4. Finally, the OPLS3 force field^17^ was applied during restrained minimizations of the IRE1 dimer and tetramer structures to refine the protein geometries.

### Protein–protein docking

To derive models of the human IRE1 tetramer, protein–protein docking was performed using SymmDock (https://bioinfo3d.cs.tau.ac.il/SymmDock/php.php)^18,19^; a symmetric docking method. The choice of the docking approach is supported by the unconventional XBP1 cleavage mediated by IRE1 model that involves one RNA stem loop hydrolysis per IRE1 RNase dimer^3,7^ (Fig. 1A). For the docking we used the prepared IRE1 back-to-back dimer (PDB ID: 4YZC) with excluded co-crystallized staurosporine in the kinase pockets, as asymmetric unit with symmetry order C2.

**Figure 2:**
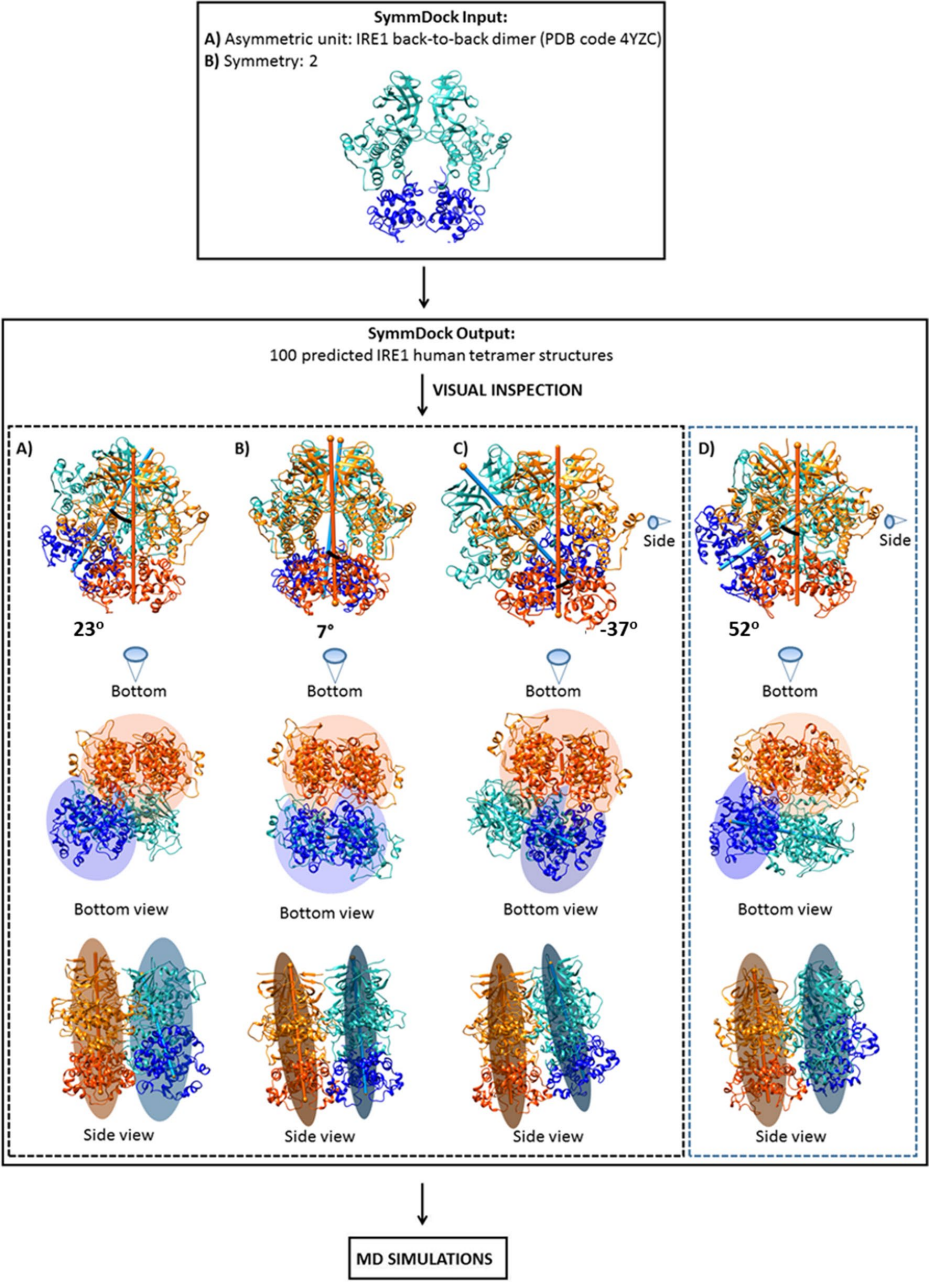
Schematic representation of the protein–protein docking scheme used for the prediction of human IRE1 tetrameric model structures. (**A**) *h*IRE1_4_(R), (**B**) *h*IRE1_4_(S), (**C**) *h*IRE1_4_(L), and (**D**) yeast crystallographic structure *y*IRE1_4_ obtained from the yeast oligomeric structure (PDB code: 3FBV^5^). The kinase domains of the dimers are shown in orange and light green and the RNase domains in red and blue, respectively. The cones specify the relative IRE1 tetramer model perspectives from the side and bottom. Protein images produced using UCSF Chimera 1.14, https://www.cgl.ucsf.edu/chimera.

First, the ability of the docking server to reproduce the native human back-to-back IRE1 dimer (PDB ID: 4YZC) was checked. Starting from the crystallographic structure, we split the dimer into monomers and subjected one monomer to protein–protein docking with SymmDock^18^. Secondly, SymmDock was used to reproduce the yeast IRE1 tetramer. Given the successful outcome of the initial benchmarking in reproducing experimental binding modes, we were confident in using this to identify different human IRE1 tetramer structures (Fig. 2). The derived tetramers [symmetrical, rotated to the right and rotated to the left; hereafter referred to as *h*IRE1_4_(S), *h*IRE1_4_(R) and *h*IRE1_4_(L), respectively] were further subjected to classical Molecular Dynamics (MD) simulations.

### Molecular dynamics simulations

For the MD simulations, a series of steps were performed:

- *System preparation*: Systems include the experimental human IRE1 dimer and yeast tetramer structures (PDB codes: 4YZC and 3FBV, respectively) and the predicted human IRE1 tetramers (“Protein–protein docking”). Each system was prepared separately, as discussed in “Selection and preparation of IRE1 crystal structures”.
- *MD simulation protocol*: Using the GROMACS 5.1 package^20^, MD simulations were performed with the AMBER14SB force field for the protein^21^ and derived parameters for phosphoserine^22^. The systems were explicitly solvated using cubic water boxes with TIP3P water^23^, under periodic boundary conditions. The cell borders were placed at least 10 Å away from the nearest protein atom, giving a total of 154,736; 145,649; 185,037; and 166,761 atoms in *y*IRE1_4_, *h*IRE1_4_(R), *h*IRE1_4_(S), and *h*IRE1_4_(L) simulation box, respectively. The systems were first neutralized and additional Na^+^/Cl^−^ counter ions added to give a physiological salt concentration of 0.154 M. All simulation runs consisted of (i) energy minimization until the force was less than 1000 kJ mol^−1^ nm^−1^, (ii) 200 ps NVT equilibration to raise the temperature to 300 K, (iii) then followed by 200 ps equilibration and (iv) 600 ns of classical molecular dynamics simulation under NPT conditions. The initial 100 ns of the sampling time were discarded as equilibration. In all simulations, the temperature was kept at 300 K by a velocity rescaling thermostat^24^ with a coupling constant of 0.1 ps. Except for the NVT pre-simulation steps, the pressure was kept at 1.01325 bar using the Parrinello-Rahman barostat^25^ with a coupling time of 5.0 ps. Using the LINCS algorithm^26^, constraints were applied on all bonds. Electrostatic forces were evaluated with the particle-mesh Ewald^27^ algorithm using a real-space cutoff of 8 Å, and van der Waals forces truncated at 8 Å and long-range corrections added. The leap-frog algorithm^28^ was employed in all the simulations, using a time step of 2 fs.

The structural information obtained during the MD simulation was analyzed in terms of root mean square deviation (RMSD), number of distinct hydrogen bonds at the dimer-dimer interface, distances between dimer centers of mass (COM) and COM distances between the two dimeric RNase sites, energy terms such as electrostatic (Ele) and van der Waals (vdW) interactions with built-in analysis tools in the GROMACS 5.1 package^20^. Free energy analysis (kcal/mol) for each of the systems were performed using an MMGBSA protocol implemented in AmberTools^29^. To analyze the angle between the dimer interface the dihedral angle between the dimer axes passing through the COM of each dimer (Supplementary Fig. S2) was measured during the 600 ns classical MD simulation. Interface dimer-dimer atomic contacts were computed using the GetContacts analysis tool (at https://getcontacts.github.io/). Modules available in GROMACS^20^ were used to perform principal components analysis (PCA)^30,31^, and modules available in AmberTools^32^ were utilized to evaluate the number of distinct hydrophobic contacts^33,34^ at the dimer-dimer interface. Low mode vibrational sampling within Schrödinger engine^35^ was used to investigate possible biologically relevant motions of the tetramer systems and obtain characteristic frequencies of the simulated tetramers. For the normal mode calculations we used the same initial tetramer structures as for the MD production runs, i.e., after the energy minimization and equilibration steps.

### XBP1 3D structure prediction

In order to predict the XBP1 three-dimensional structure, secondary structure predictions of conserved bifurcated stem-loop (BSL) sequences were performed using the MC-Fold webserver^36^. Using the RNA sequence as input, MC-Fold predicts a manifold of secondary structures as output. The tertiary structure is modelled using MC-Sym program^36^, based on the output generated by MC-Fold. Lists of tertiary structures are generated as output, minimized using the Tinker molecular modeling package^37,38^ with a steepest-descent method and the Amber-99 force-field^39^.

## Results and discussion

### Protein–protein docking analysis

To derive human IRE1 tetramer models, we used protein–protein docking. We chose the protein–protein docking server SymmDock^18^, as we expect the tetramers to display twofold symmetry^3^. As unit structure, we used the human IRE1 back-to-back dimer crystallographic structures (PDB ID: 4YZC).

We first verified that the protein–protein docking server is able to reproduce the human IRE1-back-to-back dimer structure. Starting from the human IRE1 back-to-back crystallographic structures (PDB code: 4YZC), we split the dimer into monomers and subjected two copies of one monomer to protein–protein docking with SymmDock^18^, which resulted in the best-RMSD docking pose being highly superimposable with the crystallographic one (Supplementary Fig. S3). The Cα root-mean-square deviation (rmsd) of the best-RMSD pose to the crystal structure is 1.48 Å.

Secondly, we performed another docking run; starting from the *y*IRE1_4_ (PDB ID: 3FBV) we split the tetramer into dimers and subjected two copies of one dimer to protein–protein docking with the same setup used to reproduce the human IRE1-back-to-back dimer structure. The best-RMSD docking pose is highly superimposable with the crystallographic one (Supplementary Fig. S4) with the Cα rmsd computed against the crystal structure of 1.52 Å. This indicates that the chosen protein–protein docking approach was reliable and could be applied to generate human IRE1 tetramer models.

Next, we used the human IRE1 back-to-back dimer to build a series of IRE1 tetramer structures (Fig. 2, Supplementary Figs. S5, S6, and S7). From the top 100 scored docking poses obtained by SymmDock, we selected three distinct human IRE1 tetramer configurations/conformations ranging from the three different highest ranked human IRE1 tetramer structures that are compatible with an arrangement able to connect the functional domains of IRE1 to the transmembrane region (*h*IRE1_4_(S), *h*IRE1_4_(R) and *h*IRE1_4_(L), respectively). Comparison of the tetramer models to the yeast crystallographic structure *y*IRE1_4_ (Fig. 2, Supplementary Figs. S5, S6, S7, S8, S9) show that *h*IRE1_4_(R) adopts a conformation that highly resembles *y*IRE1_4_, whereas the two other models display distinctly different conformations (Fig. 2). Noteworthy, sequence similarity and sequence identity analysis of human IRE1 revealed that the primary sequence of the cytosolic domain of yeast IRE1 has ∼ 40% sequence identity and ∼ 60% sequence similarity compared with that of *h*IRE1. To provide insights into which human tetramer form potentially has a biological role, we subjected the three human tetramer models and the yeast tetramer to 600 ns MD simulations. Prior to this analysis, we validated the quality of the three distinct human IRE1 tetramer configurations/conformations with a novel method for model quality estimation^40^. The three human IRE1 tetramer models generated have Global Scores^40^ of 0.65 ± 0.05 for *h*IRE1_4_(S) and *h*IRE1_4_(R), and 0.66 ± 0.05 for *h*IRE1_4_(L) which can be classified as predictor of correctly modelled molecular systems^40^, and is in agreement with the Global Score obtained for the *y*IRE1_4_ system (i.e. 0.65 ± 0.05).

### MD analysis

**Figure 3:**
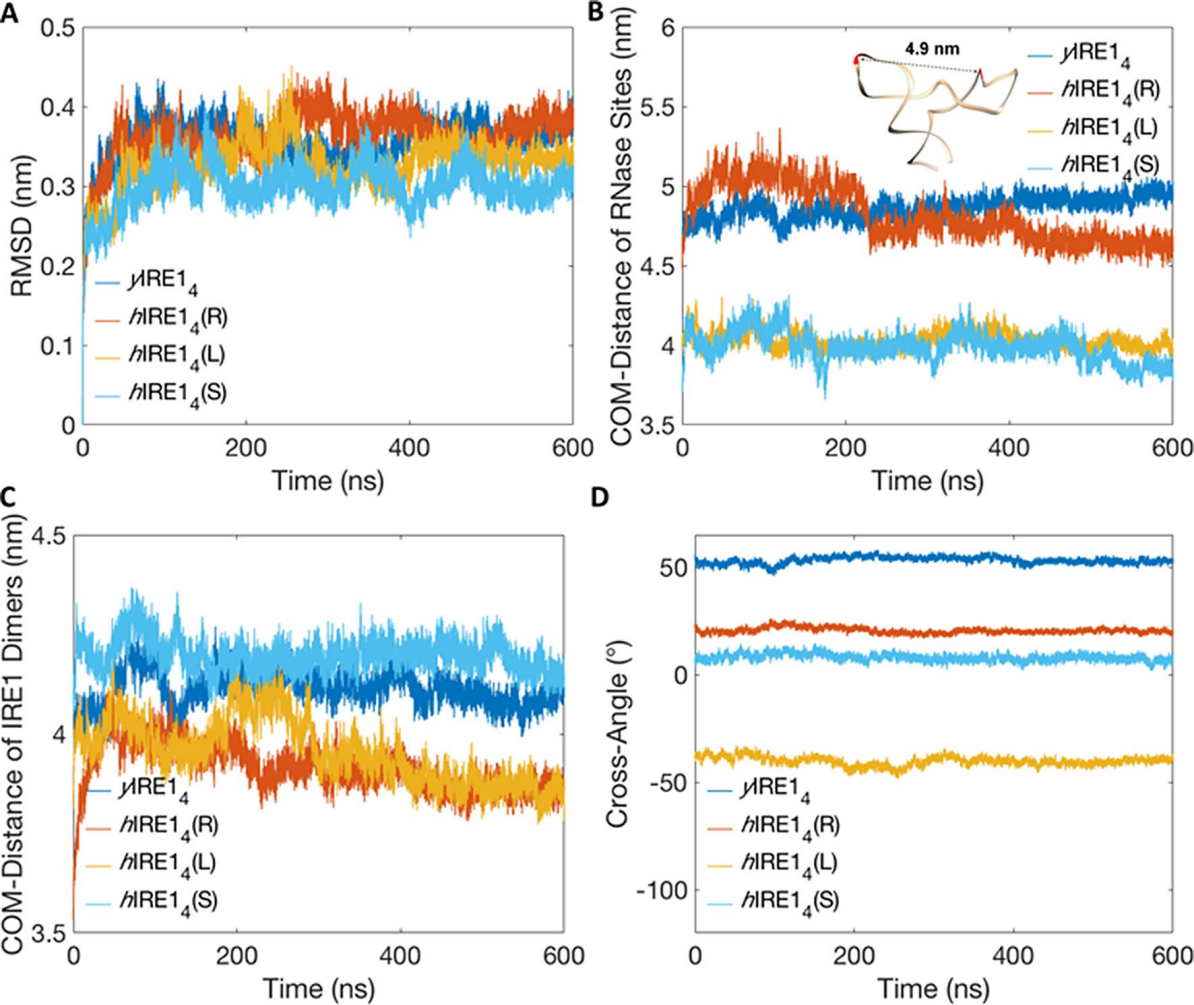
Structural data from the 600 ns MD simulations. (**A**) RMSDs of IRE1 tetramer Cα atoms of *y*IRE1_4_, *h*IRE1_4_(R), *h*IRE1_4_(L), and *h*IRE1_4_(S). (**B**) Evolution of center of mass distances between the two dimeric RNase sites. Three-dimensional structure prediction of human XBP1 mRNA and distance of the centroids of the IRE1 cleavage sites are shown. (**C**) Evolution of the distances between centers of mass of the two dimers. (**D**) Evolution of the cross-angle between the dimer interfaces (see Supplementary Fig. S2 for the definition of the cross-angle).

To assess the stability of the molecular dynamics simulations of the three human IRE1 tetramer models and the yeast IRE1 tetrameric structure, we calculated the evolution of the root-mean-square deviation (RMSD) (Fig. 3A), with respect to the initial minimized and equilibrated system. As seen in Fig. 3A, the low and relatively constant RMSD values of the trajectories indicate high stability and no major fluctuations over the 600 ns time period. Each system converges after 100 ns; therefore, our analysis will be focused on the 100–600 ns time window.

To further explore the dynamic features of the tetramer systems, we analyzed the center of mass (COM) distance between the RNase domains of the two dimers (Fig. 3B), as well as the COM distance between the two dimers (Fig. 3C). In addition, we monitored the cross-angle between the dimer interfaces (Fig. 3D). The average cross-angles are 53.2° ± 1.6°, 20.5° ± 1.3°, − 40.2° ± 2.1°, and 8.0° ± 1.6° for the yeast IRE1 tetramer, and the three human IRE1 tetramer models: *h*IRE1_4_(R), *h*IRE1_4_(L), and *h*IRE1_4_(S), respectively (Fig. 3D and Supplementary Fig. S10). The *h*IRE1_4_(R) and the *y*IRE1_4_ systems show RNase dimer center of mass (COM) distances around 4.8–5.0 nm while *h*IRE1_4_(L) and *h*IRE1_4_(S) display distances within 4.0–4.2 nm (Fig. 3B and Supplementary Fig. S11). For *h*IRE14(R) (Supplementary Fig. S11) we observe a bimodal distribution with the higher COM distance during the 0–200 ns and smaller COM distance during 200–600 ns. These COM-distances and cross-angles may have a large impact on the molecular mechanism of XBP1 mRNA binding to the IRE1 tetramer and subsequent catalytic splicing. For this reason, we built an XBP1 3D structure model which allows us to correlate the distance between the centroids of the IRE1 cleavage sites with the evolution of center of mass distances between the two dimeric RNase sites. The cleavage sites are placed ~ 4.9 nm apart which agrees with the center of mass distances between the two dimeric RNase sites of *y*IRE1_4_ and *h*IRE1_4_(R), while it is incompatible with the *h*IRE1_4_(L) and *h*IRE1_4_(S) dimeric RNase sites centers of mass.

**Figure 4:**
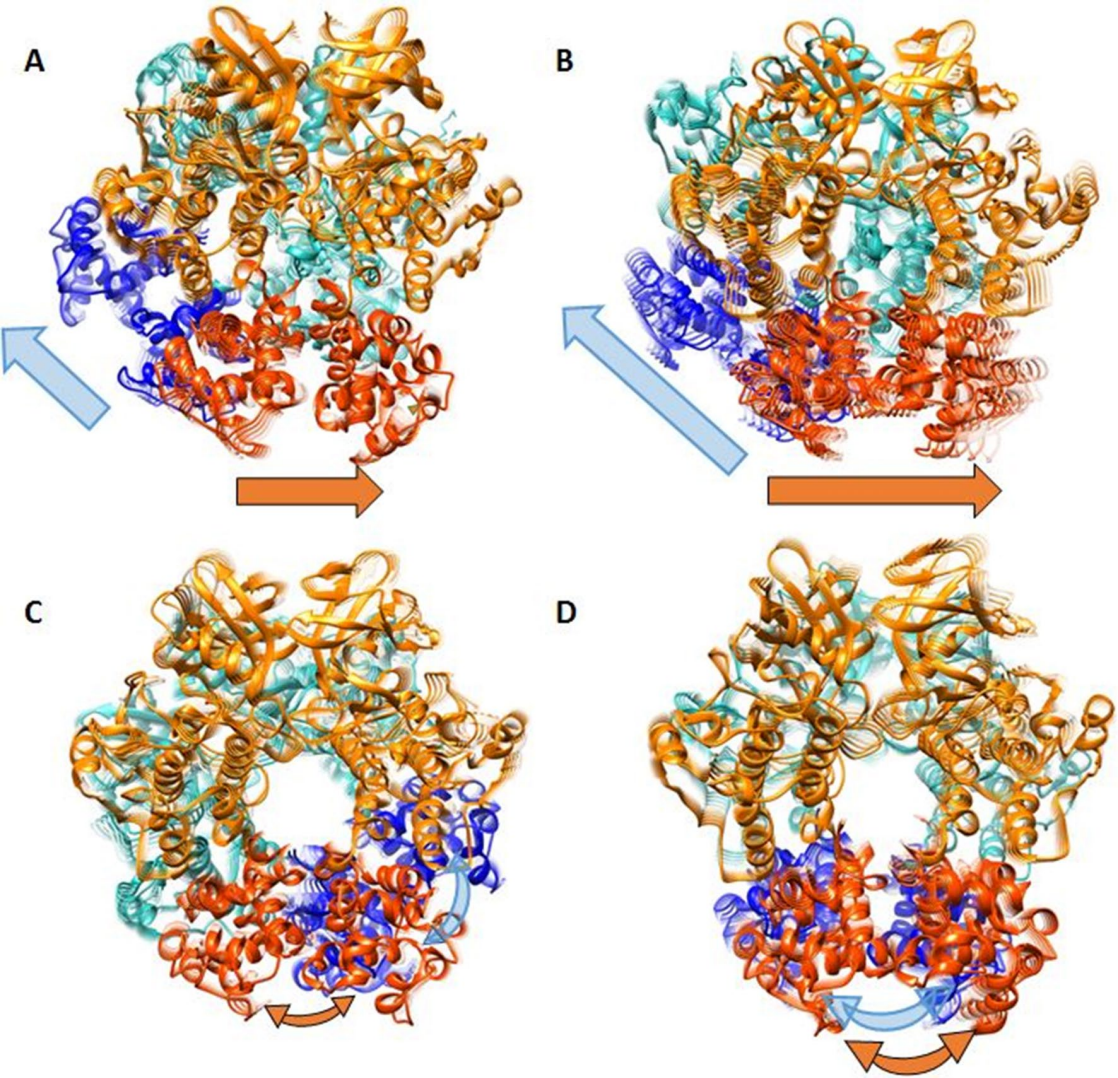
Motion of PC1 PCA obtained from MD simulations of IRE1 heavy atoms of (**A**) *y*IRE1_4_ and human IRE1 tetramer models: (**B**) *h*IRE1_4_(R), (**C**) *h*IRE1_4_(L), and (**D**) *h*IRE1_4_(S). The straight arrows indicate IRE1 RNase region breathing expressed as an opening of each dimer in the RNase domain while the curved arrows describe tilting motion within each RNase domain. The kinase domains are shown in orange and light green and the RNase domains in red and blue, respectively. Protein images produced using UCSF Chimera 1.14, https://www.cgl.ucsf.edu/chimera.

To capture the predominant motions of the IRE1 tetramers that could be essential for IRE1 RNase splicing activity, we performed principal component analyses (PCA)^30,31^ of the trajectories. The most dominant motions in the MD simulations are represented within principal components 1 and 2, which accounts for 30–45% of the total variance, with the first component being by far the most prominent (Supplementary Fig. S12). As seen in Supplementary Videos S1, S2, along PC1 and PC2 *y*IRE1_4_ samples regions corresponding to an “open” tetramer conformation with extended RNase domains (Fig. 4A, Supplementary Fig. S13A and Supplementary Video S1, S2). As illustrated in Fig. 4A, the motion of PC1 consists of an opening of the RNase domain of each dimer while PC2 (Supplementary Fig. S13A) corresponds to one dimer tilting motion and one monomer of the other dimer opening the RNase domain. The observed dynamics and flexibility of the RNase domains, we believe is important for the catalytic splicing activity of IRE1.

For *h*IRE1_4_(R), and to a lesser extent, for *h*IRE1_4_(L), the conformational regions explored in PC1 and PC2 are comparable with those for *y*IRE1_4_, i.e. an “opening” of the tetramer and extension of RNase domains (Fig. 4B and Supplementary Fig. S13B, Supplementary Videos S3, S4 for *h*IRE1_4_(R) and Fig. 4C and Supplementary Fig. S13C, Supplementary Videos S5 and S6 for *h*IRE1_4_(L), respectively). PC1 for *h*IRE1_4_(R) is a pure opening of the RNase domain of each dimer while PC2 describes the two dimers tilting motion in opening the RNase domain. For *h*IRE1_4_(L) PC1 and PC2 both include tilting of the two dimers in opening the RNase domain. The *h*IRE1_4_(S) model exhibit a different set of large-scale collective motions in which PC1 corresponds to a “compressed” RNase conformation driven by tilting of the two RNase dimers (Fig. 4D, Supplementary Video S7) and PC2 corresponds to an “extended” RNase conformation as for previous systems driven again by tilting of the two RNase dimers. (Supplementary Fig. S13D and Supplementary Video S8). Sampling of the low mode vibrational motions revealed a similar movement, i.e. an oscillation between open and compressed conformations of RNase domains, in all four tetramers investigated. These low mode vibrational sampling motions are in qualitatively good agreement with the PCA movement displaying the same dynamic behaviour (Supplementary Fig. S14). The time frame of the described motions, calculated by inversion of the normal frequencies, suggests that the low mode vibrational motions occur within the ~ 300 ns time period range (Supplementary Fig. S15), which justifies the selected MD simulation times (i.e. 600 ns).

Finally, to predict which tetramer configurations that are energetically most favorable and thus likely to have a biological role we estimated the free energies of the tetramers. As seen in Table 1, both MMGBSA and MMPBSA interaction energies^29^ imply a significant stabilization of the *y*IRE1_4_ and *h*IRE1_4_(R). The *h*IRE1_4_(L) and *h*IRE1_4_(S) models exhibit positive MMGBSA interaction energies, indicating less stable tetramer complexes.

**Table 1:** Free energy analysis (kJ/mol) for (A) *y*IRE1_4_, (B)* h*IRE1_4_(R), (C) *h*IRE1_4_(L), and (D) *h*IRE1_4_(S).

Tetramer	MMGBSA	MMPBSA
(A) *y*IRE1_4_	− 206.50 (75.43)	− 445.68 (110.30)
(B) *h*IRE1_4_(R)	− 171.33 (59.44)	− 441.87 (80.87)
(C) *h*IRE1_4_(L)	73.26 (62.37)	− 240.11 (71.20)
(D) *h*IRE1_4_(S)	277.78 (88.32)	458.45 (206.33)

Numbers in parentheses present the standard deviations.

**Figure 5:**
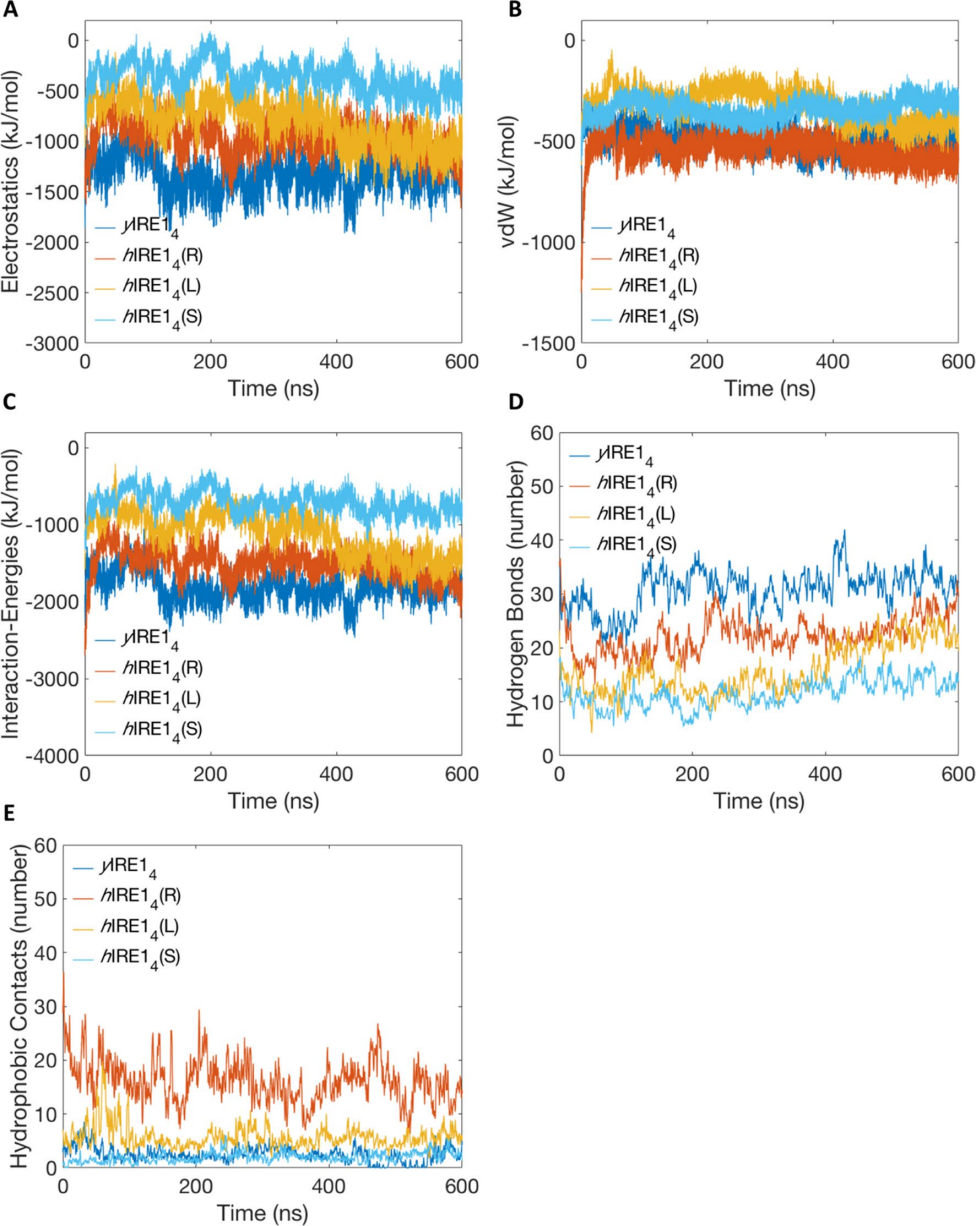
Evolution of interaction energy profiles showing the (**A**) electrostatics energies, (**B**) vdW-energies and (**C**) total interaction-energies for dimer A with dimer B during the MD simulations of *y*IRE1_4_, *h*IRE1_4_(R), *h*IRE1_4_(L) and *h*IRE1_4_(S). (**D**) Hydrogen bonds between dimer pairs. (**E**) Hydrophobic contacts at the dimer-dimer interface.

We also monitored the time evolution of interaction energies using the GROMACS analysis tools (Fig. 5A–C), which showed a similar trend compared to MMGBSA/MMPBSA analysis. Both *y*IRE1_4_ and *h*IRE1_4_(R) are more energetically favorable, despite that* h*IRE1_4_(L) moves to more stable interactions towards the end of the trajectory. The *h*IRE1_4_(S) model exhibits the most unfavorable/almost repulsive electrostatic energies (Fig. 5A). The trends in interaction energies are coupled to the number of hydrogen bond interactions and hydrophobic contacts between the dimers (Fig. 5D,E). The number of H-bonds represents the trend in electrostatic energies, where a higher number of H-bonds occur for *y*IRE1_4_ and *h*IRE1_4_(R) compared to the other two (Fig. 5D). The *h*IRE1_4_(R) model also exhibits slightly more negative vdW-interaction energies compared to the other tetramers, which is evidenced by the larger number of hydrophobic contacts between the dimers in this case (Fig. 5E).

**Figure 6:**
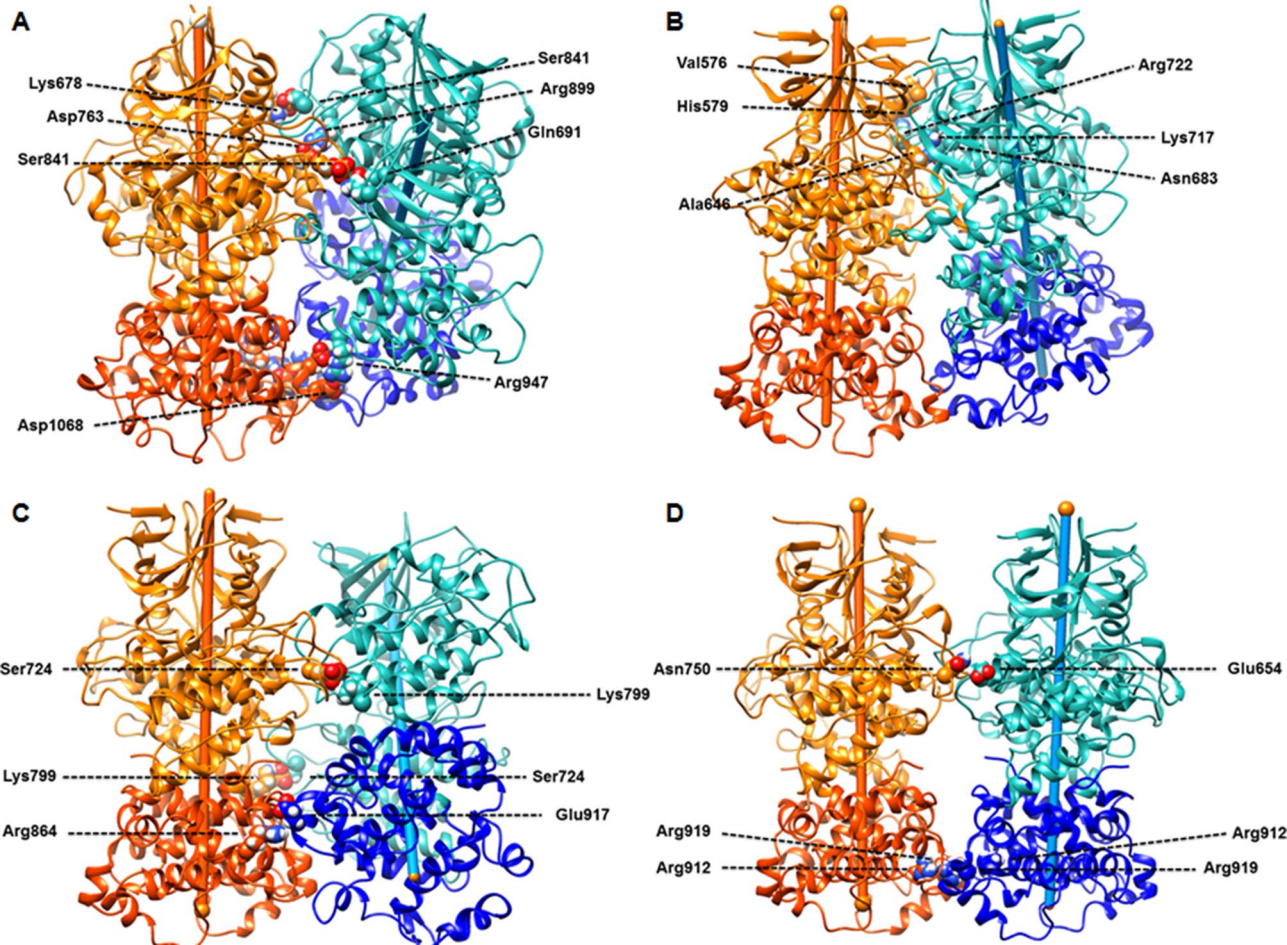
Contacts between dimer A with dimer B with highest frequency during the MD simulations of (**A**) *y*IRE1_4_, (**B**) *h*IRE1_4_(R), (**C**) *h*IRE1_4_(L), and (**D**) *h*IRE1_4_(S) are shown. The kinase domains are shown in orange and light green and the RNase domains in red and blue, respectively. Protein images produced using UCSF Chimera 1.14, https://www.cgl.ucsf.edu/chimera.

To obtain further structural insights into the differences in interaction energies for the tetramer models, we investigated the interface dimer-dimer atomic contacts between the systems (Fig. 6 and Supplementary Figs. S16–S19). Lists of all contacts observed in the different tetramers are shown in Supplementary Figs. S16–S19. The contacts present for 100% of the trajectories are shown in Fig. 6. The hIRE1_4_(S) model forms a number of repulsive contacts between arginines in the RNase domains, which explains the trend in electrostatic energies. Over the course of the 600 ns MD simulations *y*IRE1_4_ and *h*IRE1_4_(R) exhibit a larger number of contacts compared to *h*IRE1_4_(L) (Supplementary Figs. S16–S19). This data explains the higher relative stability of *h*IRE1_4_(R) compared to *h*IRE1_4_(L) and hIRE1_4_(S).

In summary, the combined analyses of interaction energies, hydrogen bonds and hydrophobic contacts, as well as the existence of “open” → “closed” tetramer RNase motions suggest that the two human IRE1 tetramer forms, *h*IRE1_4_(R) and *h*IRE1_4_(L), can co-exist. However, which of these tetramers that attains a bioactive conformation for XBP1 splicing probably depends on the COM-COM distance between the RNase domains of the dimers. Based on the COM–COM distance analysis, the *h*IRE1_4_(R) (Fig. 3C) shows a similar COM distance as *y*IRE1_4_, while the *h*IRE1_4_(L) conformer exhibits a ~ 10 Å smaller distance which, depending on the distance between the two splicing sites of the stem-loops of XBP1, can potentially interfere with the ability of the tetramer to bind XBP1 RNA.

## Conclusions and perspective

Using molecular protein–protein docking and molecular dynamics simulations, we investigated possible orientations of the human IRE1 tetramer structure (*h*IRE1_4_(R), *h*IRE1_4_(L) and *h*IRE1_4_(S)) and structurally assessed their biological relevance through analyses of 2.4 μs of all-atom molecular dynamics (MD) simulations in explicit solvent. A detailed analysis of the IRE1 dimer–dimer interactions for the tetramer systems, together with the COM distances between the RNase domains of the dimers, and characteristic macroscopic motions deduced by PCA suggests that the *h*IRE1_4_(R) model represents the most favorable configuration and should be employed for the future studies of the complex IRE1-XBP1 recognition process and mechanisms of RNA splicing. The calculated PCA and NMA dominant large scale motions in all investigated systems provide a molecular level validation of IRE1 RNase activation and IRE1 clustering as a dynamic process^3,6^. In accordance with experimental studies^4^ the current simulations corroborate the dynamics of the IRE1 tetramer, in contrast with the static picture provided by crystal structures. The IRE1 tetramer dynamics furthermore provide insight into the mechanistic assembly and disassembly of the even larger oligomers repeatedly observed in cells, as being a dynamic process rather than locked arrangements of IRE1 oligomers. The structural data indicates a IRE1 dimer-dimer interface as shown in Fig. 2, where *h*IRE1_4_(R) adopts a conformation that highly resembles *y*IRE1_4_, whereas the two other models generated herein display distinctly different conformations. This could stimulate experimentally verifiable predictions about IRE1 oligomerization in different complex topologies. In accordance with 3D structure predictions of human XBP1 mRNA^7^ and COM distances between the RNase domains of the dimers during the 600 ns MD simulations, we corroborate the previous hypothesis^7^ that at least an IRE1 tetramer is required to complete the splicing reaction of XBP1. The newly designed 3D structure prediction on human XBP1 mRNA could promote further IRE1/XBP1 computations and obtained IRE1-XBP1 recognition trends which can be validated using experimental studies. Indeed, this study emphasizes the importance of the correlated movements that combine the two IRE1 dimer RNase domains in a concerted mechanism, possibly facilitating the initial binding of XBP1, followed by the catalytic splicing. Despite the IRE1 tetramer structural complexity, the movements observed for the four systems can be classified as a breathing motion of the RNase domains, characteristic for the *y*IRE1_4_ and *h*IRE1_4_(R) models, and tilting motion within each RNase domain characteristic for the *h*IRE1_4_(L) and *h*IRE1_4_(S) models. Our data suggest that the XBP1 mRNA splicing reaction can be driven by a series of coordinated motions at the tetramer level. The designed all-atom models of the human IRE1 tetramers provide new insights into the mechanism of IRE1 molecular activation and open up for future studies of IRE1 signaling.

From this perspective, combining experimental evidence^41^ with structural data and MD simulations could advance the understanding of the role of IRE1 autophosphorylation in the IRE1 oligomerization and activation of its RNase activity. The unphosphorylated kinase/RNase domain of human IRE1 in the face-to-face dimer (PDB code: 3P23) can be studied with advanced computational methodologies as a key structural arrangement between IRE1 monomers during the trans-autophosphorylation process, and an intermediate prior to back-to-back dimer and higher-order oligomer formation^3^.

## Supplementary information

Supplementary Figures.

Supplementary Video 1.

Supplementary Video 2.

Supplementary Video 3.

Supplementary Video 4.

Supplementary Video 5.

Supplementary Video 6.

Supplementary Video 7.

Supplementary Video 8.

## Data Availability

All simulation protocols, protein–protein docking datasets and trajectory datasets are freely accessible at zenodo.org as https://doi.org/10.5281/zenodo.3920875.
