# Supplementary material for: New insights on human IRE1 tetramer structures based on molecular modeling
Source: Sci Rep. 2020 Oct 15;10:17490. doi: 10.1038/s41598-020-74347-8 (PMC7567888; doi:10.1038/s41598-020-74347-8)
Supplement: Supplementary file 1 — Supplementary Figures. [file 41598_2020_74347_MOESM1_ESM.pdf]

New insights on human IRE1 tetramer structures based on molecular modeling

Antonio Carlesso[1,2], Johanna Hörberg[1], Anna Reymer[1] and Leif A. Eriksson[1]*

[1]Department of Chemistry and Molecular Biology, University of Gothenburg, 405 30 Göteborg, Sweden

[2]Faculty of Biomedical Sciences, Institute of Computational Science, Università della Svizzera italiana (USI), Lugano, Switzerland

SUPPLEMENTARY MATERIAL

Table of Contents

**Supplemental Videos S1 – S8**	S3
**Figure S1**	S4
**Figure S2**	S5
**Figure S3**	S6
**Figure S4**	S7
**Figure S5**	S8
**Figure S6**	S9
**Figure S7**	S10
**Figure S8**	S11
**Figure S9**	S12
**Figure S10**	S13
**Figure S11**	S14
**Figure S12**	S15
**Figure S13**	S16
**Figure S14**	S17
**Figure S15**	S18
**Figure S16**	S19
**Figure S17**	S20
**Figure S18**	S21
**Figure S19**	S22

Supplemental Videos

Motions of Principal Components 1 and 2, respectively, for

y$IRE1_4$: Video_S1.avi and Video_S2.avi

h$IRE1(R)_4$: Video_S3.avi and Video_S4.avi

h$IRE1(L)_4$: Video_S5.avi and Video_S6.avi

h$IRE1(S)_4$: Video_S7.avi and Video_S8.avi

In each video the kinase domains are shown in orange and light green and the RNase domains in red and blue, respectively.

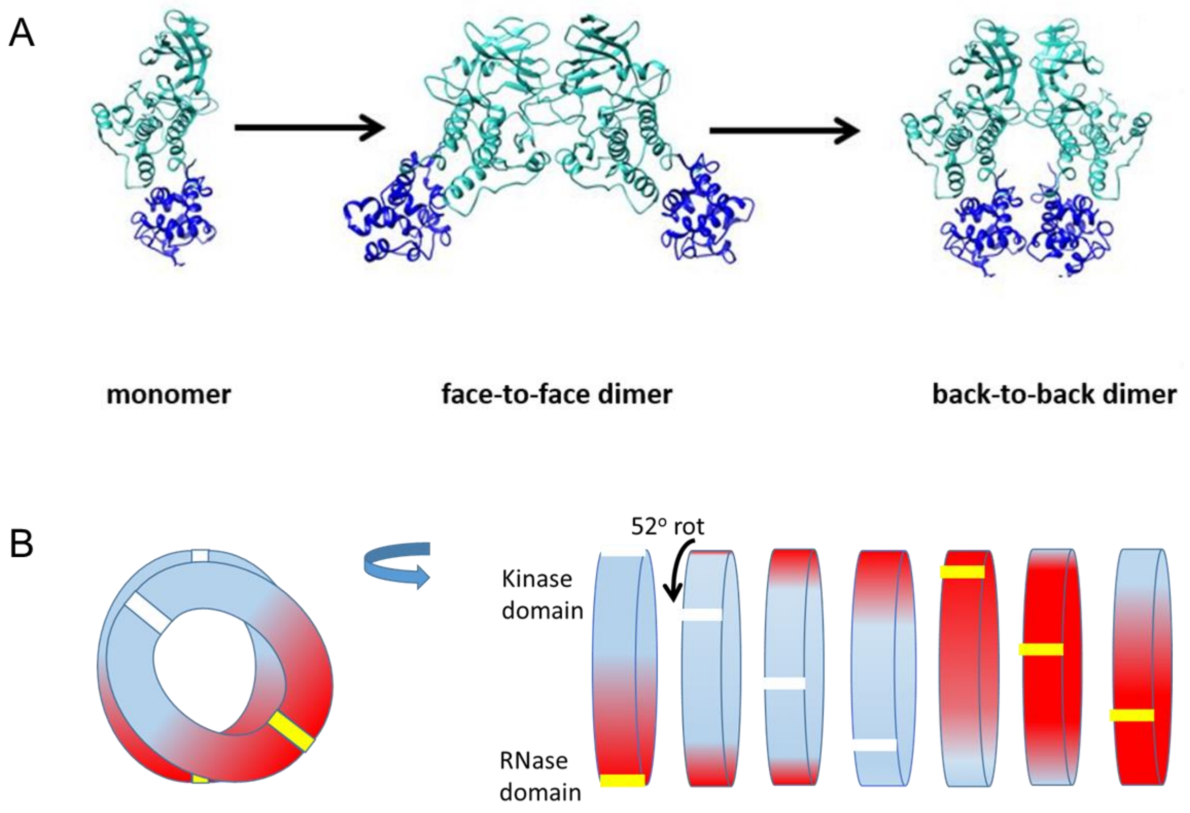

Figure S1. Overview of the structural rearrangement upon IRE1 activation. **A**. IRE1 monomer, face-to-face dimer and back-to-back dimer are shown. The kinase domain is shown in light green and the RNase domain in blue. Protein images produced using UCSF Chimera 1.14, https://www.cgl.ucsf.edu/chimera. **B**. Cartoon image of dimer pair (left) and heptameric oligomer of dimers, illustrating the 52° rotation between each consecutive dimer pair. Kinase domains in light blue, RNase domains in red.

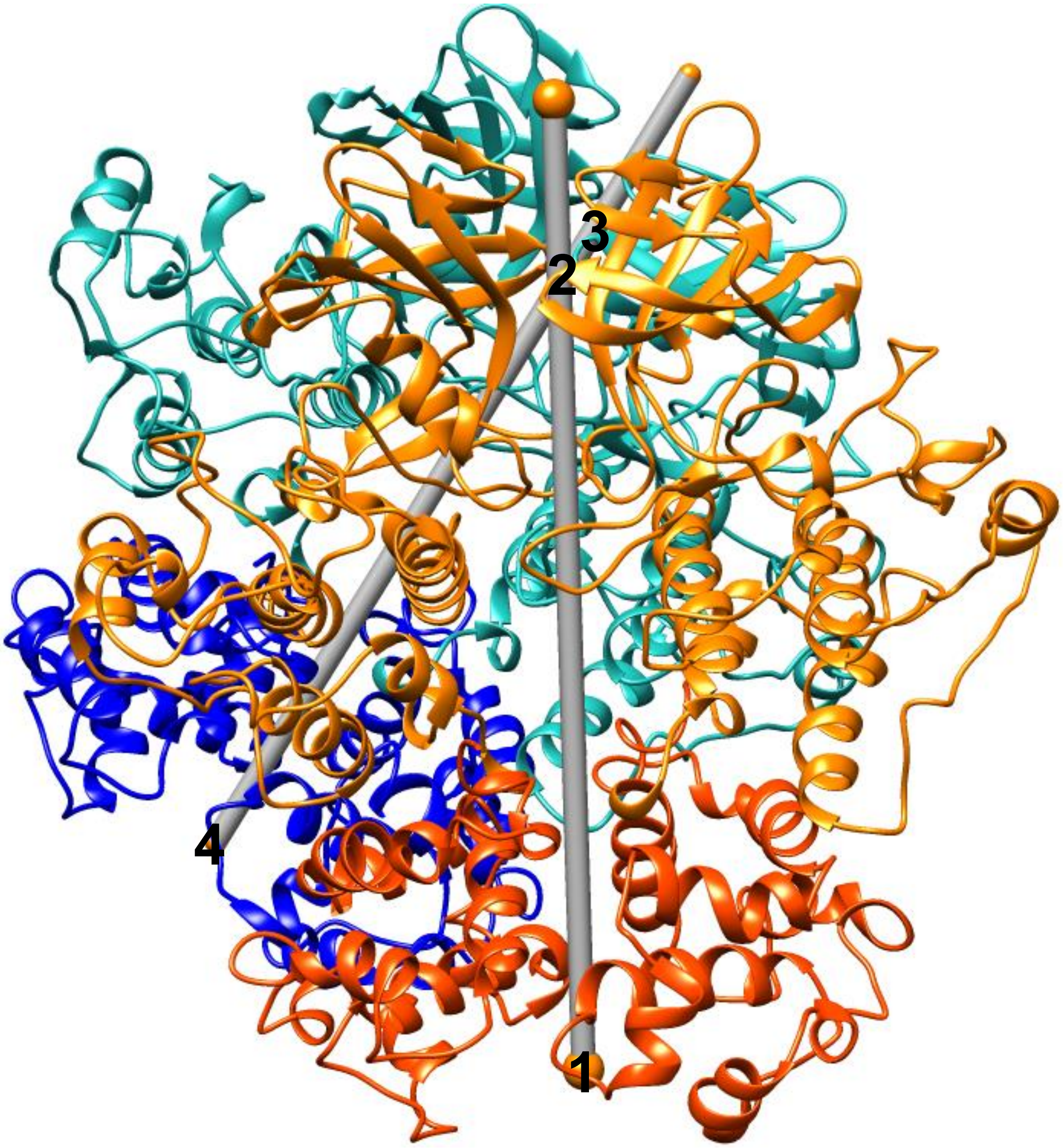

Figure S2. Schematic representation of the calculated dihedral angle between the dimer axes passing through centers of mass (COM) at the dimer interfaces. The numbers 1-2-3-4 define the dihedral (cross) angle between the dimers. The kinase domains are shown in orange and light green and the RNase domains in red and blue, respectively. Protein images produced using UCSF Chimera 1.14, https://www.cgl.ucsf.edu/chimera.

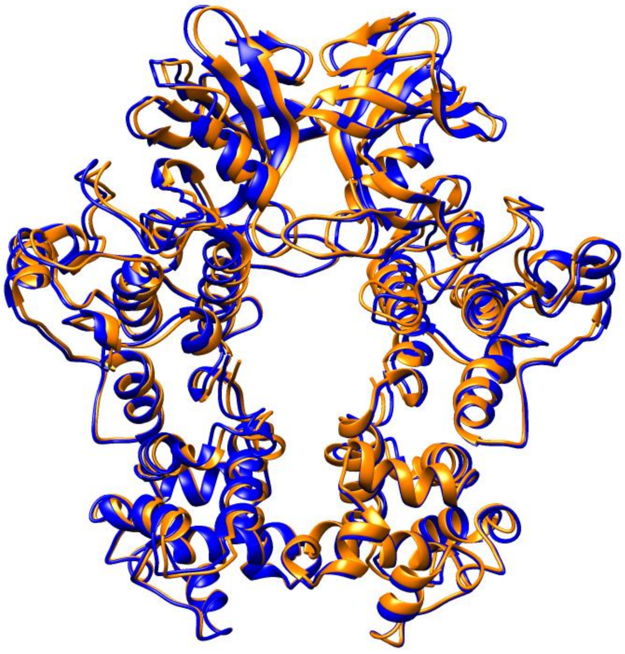

Figure S3. Superposition of the best-RMSD pose predicted by SymmDock (orange) within TOP-100 poses onto the native back-to-back crystallographic structure (PDB ID: 4YZC) (blue). The resulting RMSD value (Cα atoms) is 1.48 Å. Protein images produced using UCSF Chimera 1.14, https://www.cgl.ucsf.edu/chimera.

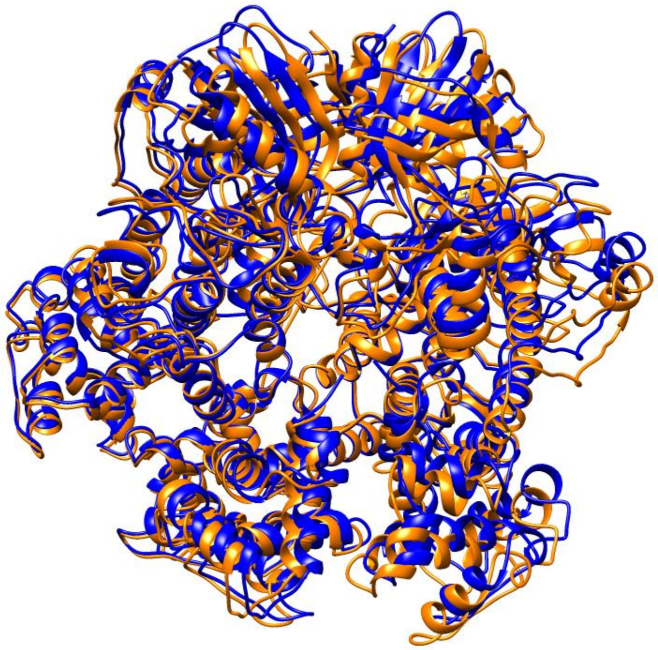

Figure S4. Superposition of the best-RMSD pose of the yeast tetramer structure predicted by SymmDock (orange) within TOP-100 poses, onto the *y*$IRE1_4$ (PDB ID: 3FBV) (blue). The resulting RMSD value (Cα atoms) is 1.52 Å. Protein images produced using UCSF Chimera 1.14, https://www.cgl.ucsf.edu/chimera.

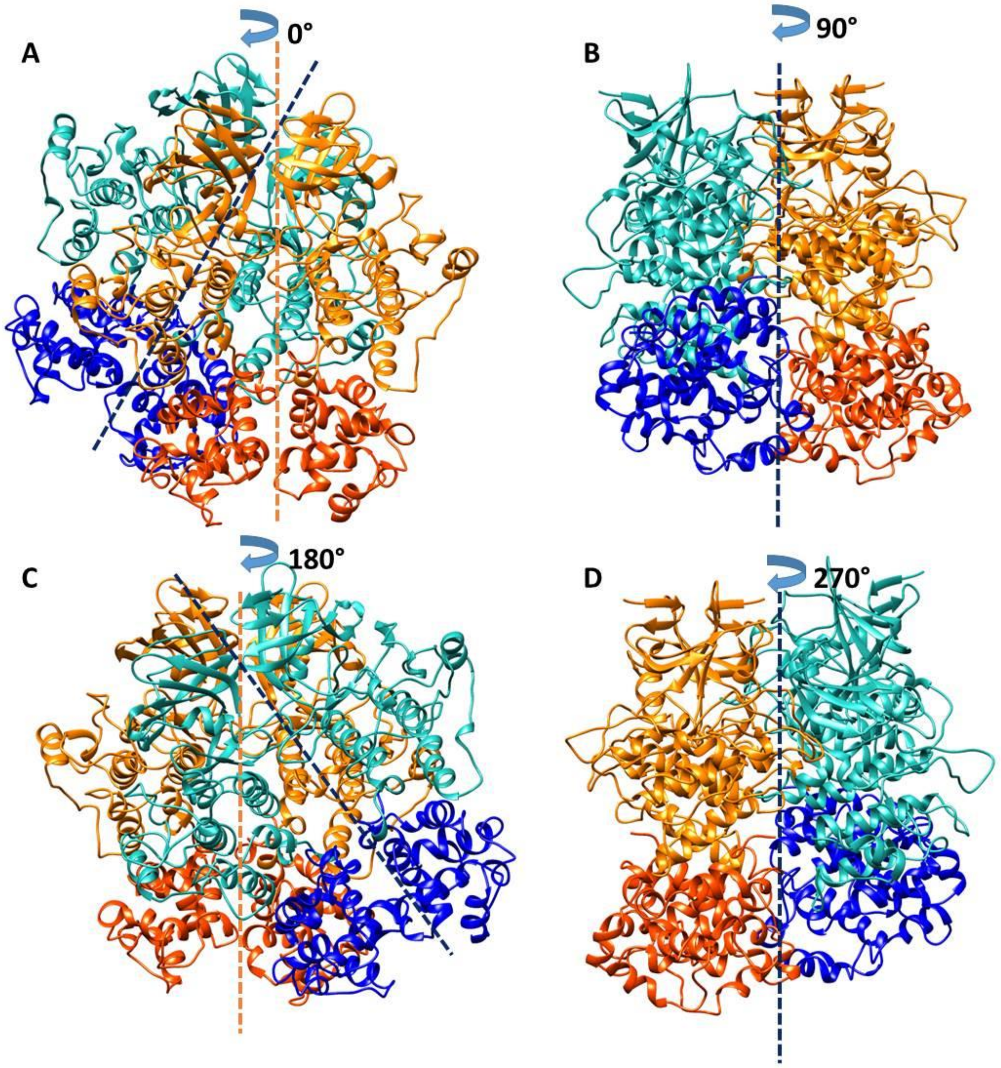

Figure S5. *h*IRE1$_4$(R) model from different viewing angles: (A) 0°, (B) 180°, (C) 90° and (D) 270°. The kinase domains are shown in orange and light green and the RNase domains in red and blue, respectively. Protein images produced using UCSF Chimera 1.14, https://www.cgl.ucsf.edu/chimera.

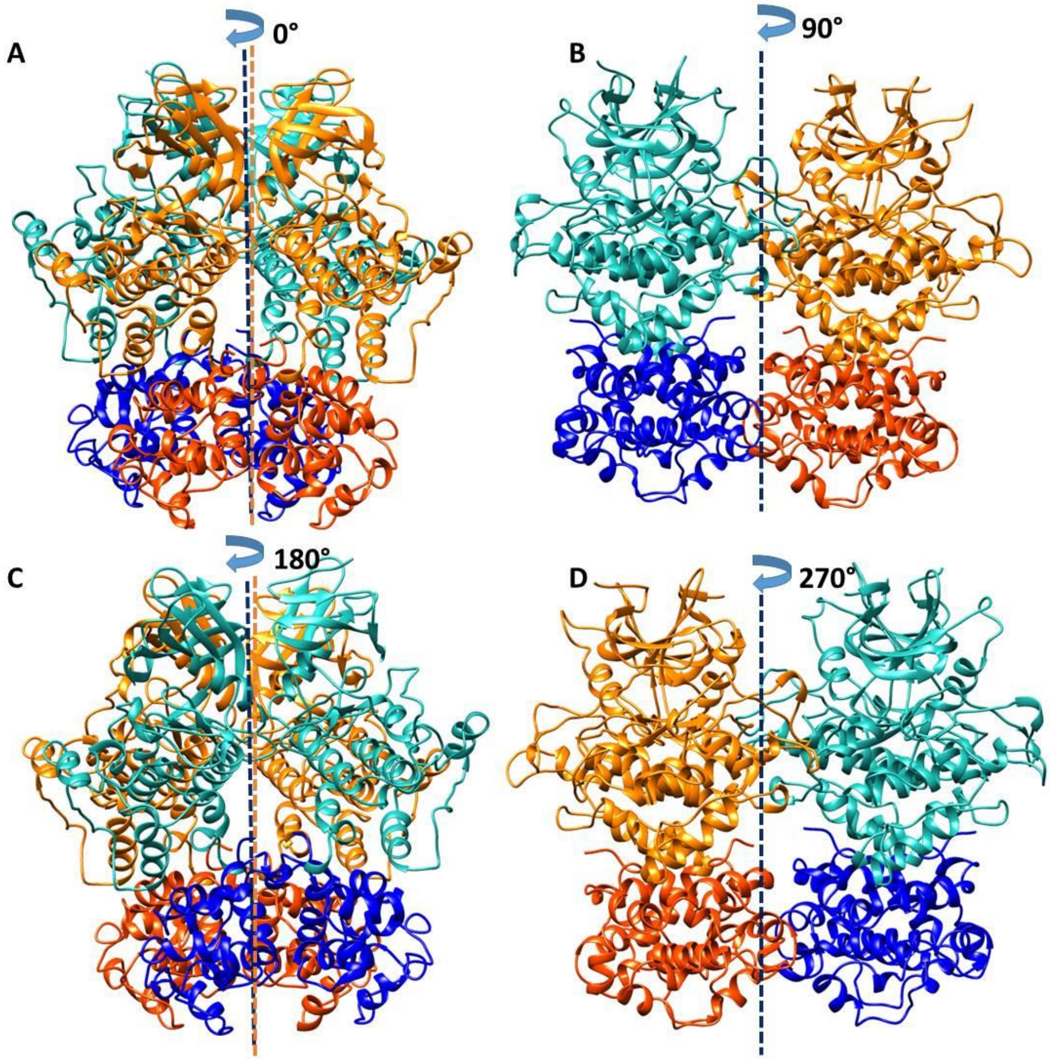

Figure S6. *h*IRE1$_4$(S) from different viewing angles: (A) 0°, (B) 180°, (C) 90° and (D) 270°. The kinase domains are shown in orange and light green and the RNase domains in red and blue, respectively. Protein images produced using UCSF Chimera 1.14, https://www.cgl.ucsf.edu/chimera.

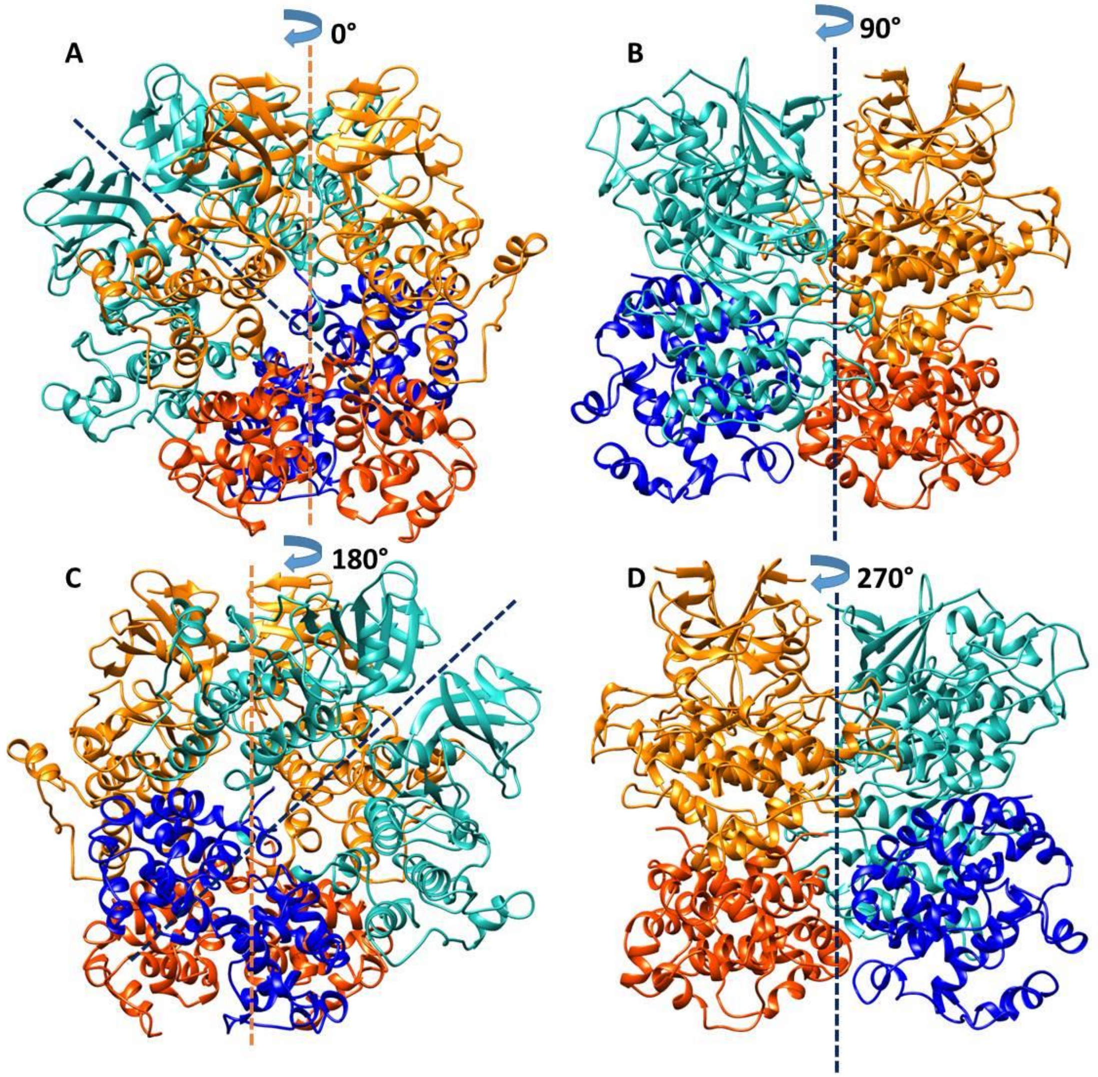

Figure S7. *h*IRE1_4(L) from different viewing angles: (A) 0°, (B) 180°, (C) 90° and (D) 270°. The kinase domains are shown in orange and light green and the RNase domains in red and blue, respectively. Protein images produced using UCSF Chimera 1.14, https://www.cgl.ucsf.edu/chimera.

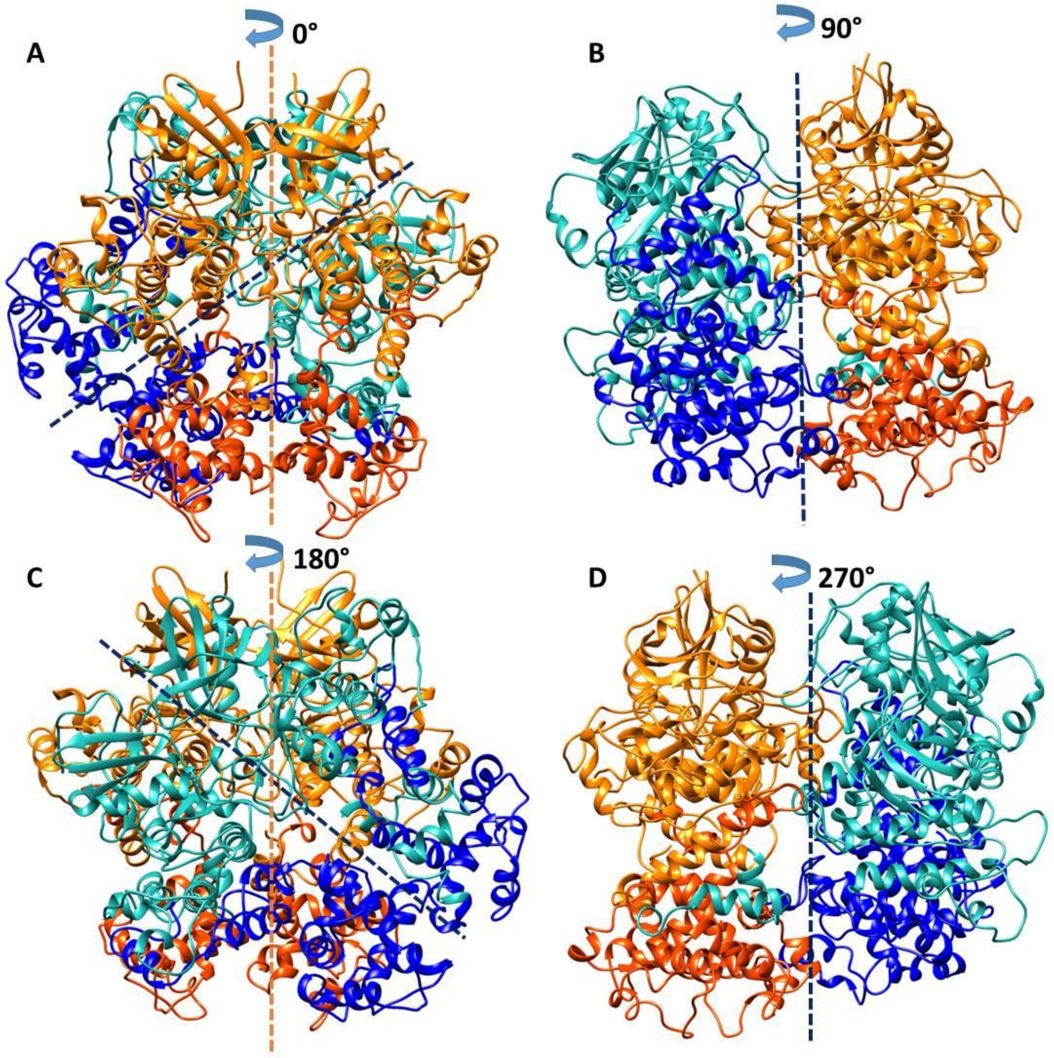

Figure S8. *y*IRE1$_4$(PDB ID: 3FBV) from different viewing angles: (A) 0°, (B) 180°, (C) 90° and (D) 270°. The kinase domains are shown in orange and light green and the RNase domains in red and blue, respectively. Protein images produced using UCSF Chimera 1.14, https://www.cgl.ucsf.edu/chimera.

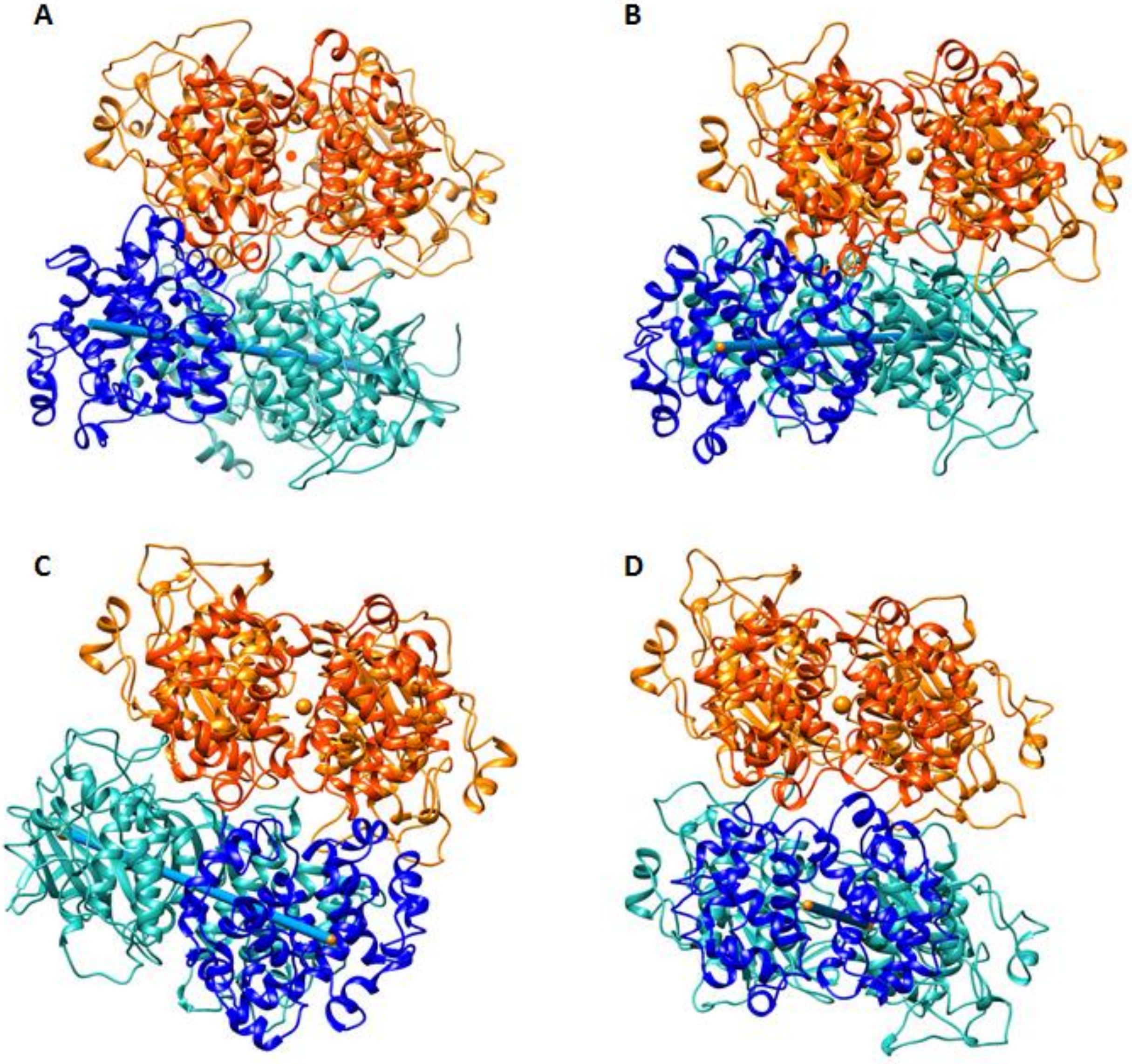

Figure S9. Zoomed-in view of the RNase domains for (A) *y*IRE1$_4$(PDB ID: 3FBV), (B) *h*IRE1$_4$(R), (C) *h*IRE1$_4$(L), and (D) *h*IRE1$_4$(S). The kinase domains are shown in orange and light green and the RNase domains in red and blue, respectively. The orientation is held fixed on the RNase domains of the orange/red dimer pair, viewed from 'bottom' (cf Figure 2 in main paper). Protein images produced using UCSF Chimera 1.14, https://www.cgl.ucsf.edu/chimera.

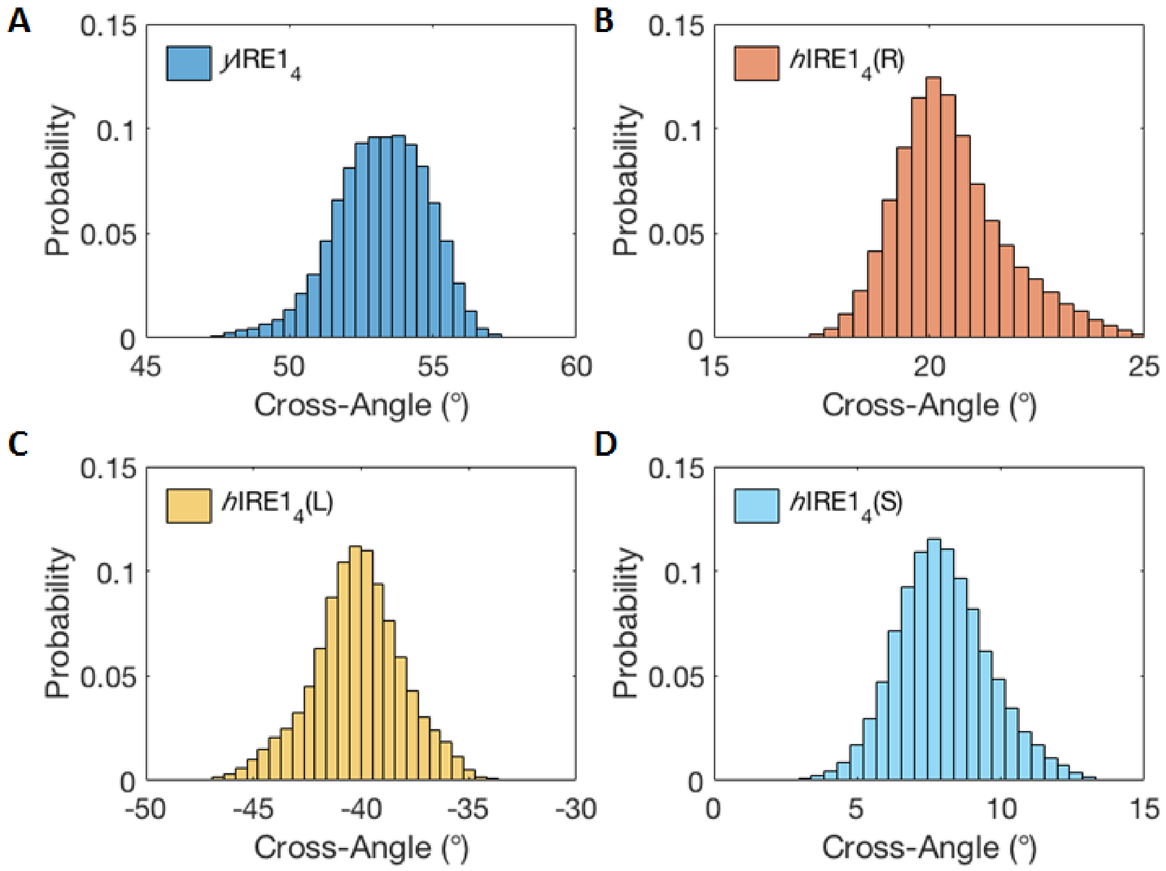

Figure S10. Cross-angle distributions between the dimers interfaces of (A) *y*IRE1$_4$, and the human IRE1 models: (B) *h*IRE1$_4$(R), (C) *h*IRE1$_4$(L), and (D) *h*IRE1$_4$(S).

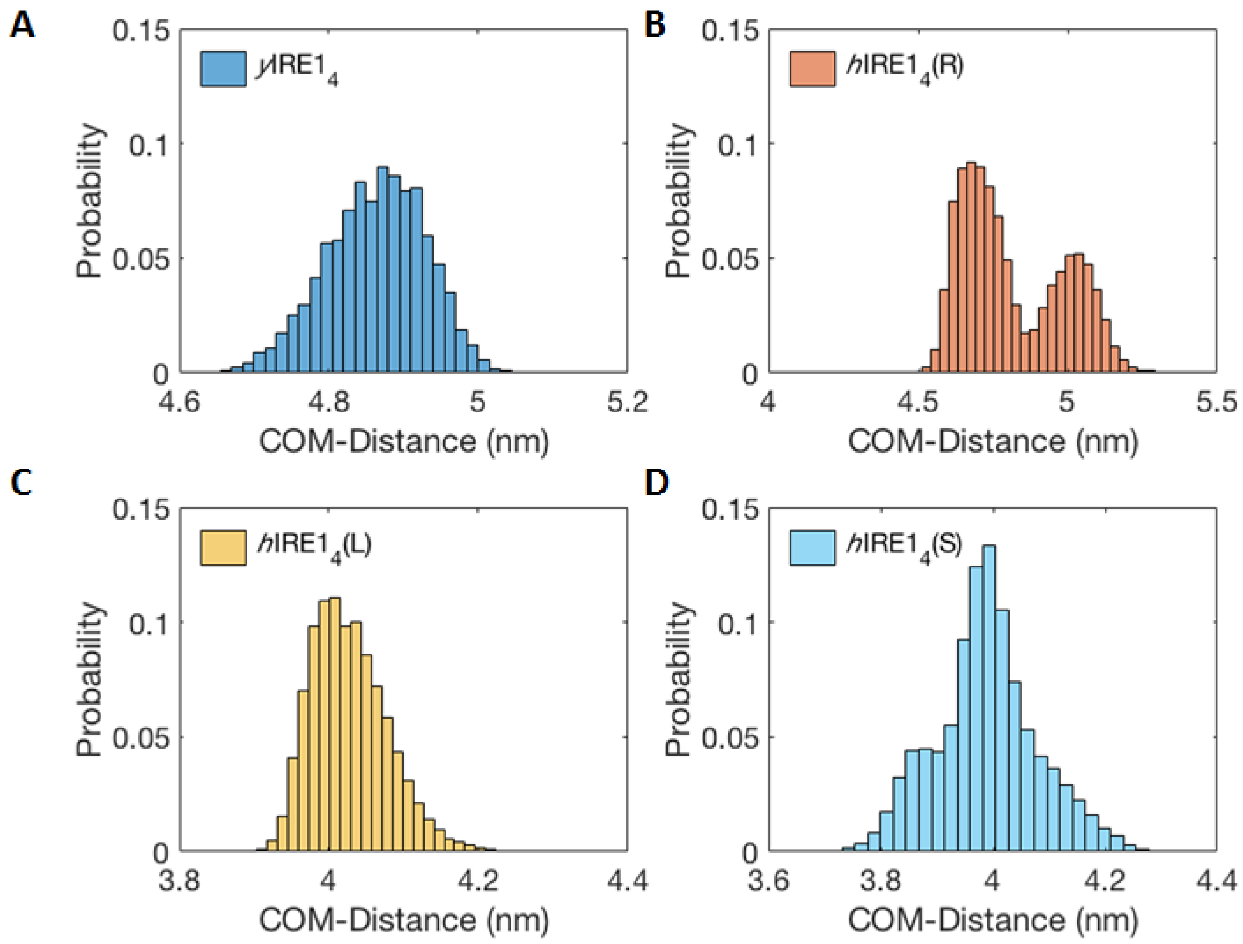

Figure S11. Center of mass distances between the two dimeric RNase sites distributions between the dimers interfaces of (A) *y*IRE1_4, and the human IRE1 models: (B) *h*IRE1_4(R), (C) *h*IRE1_4(L), and (D) *h*IRE1_4(S).

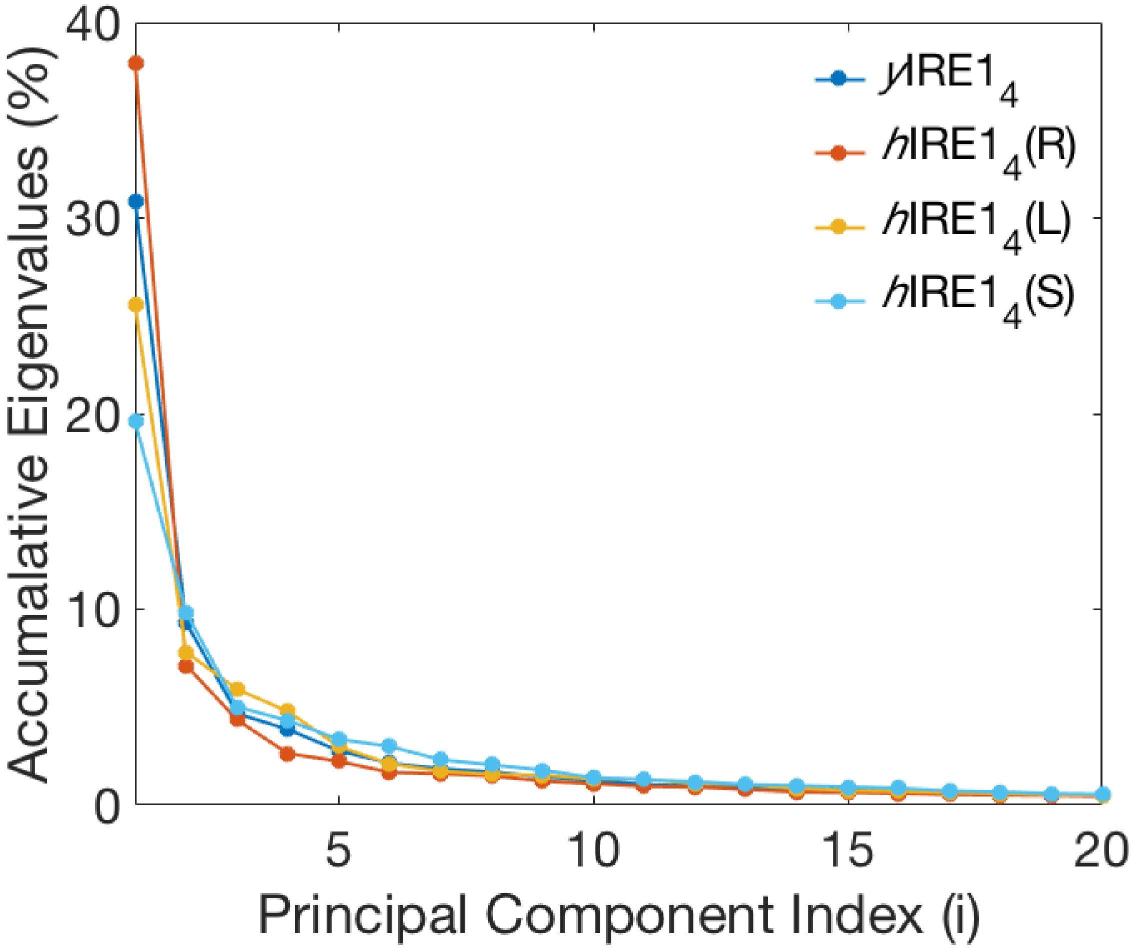

Figure S12. The variance exerted by each of the 20th first principal components from the PCA for the IRE1 tetramer systems.

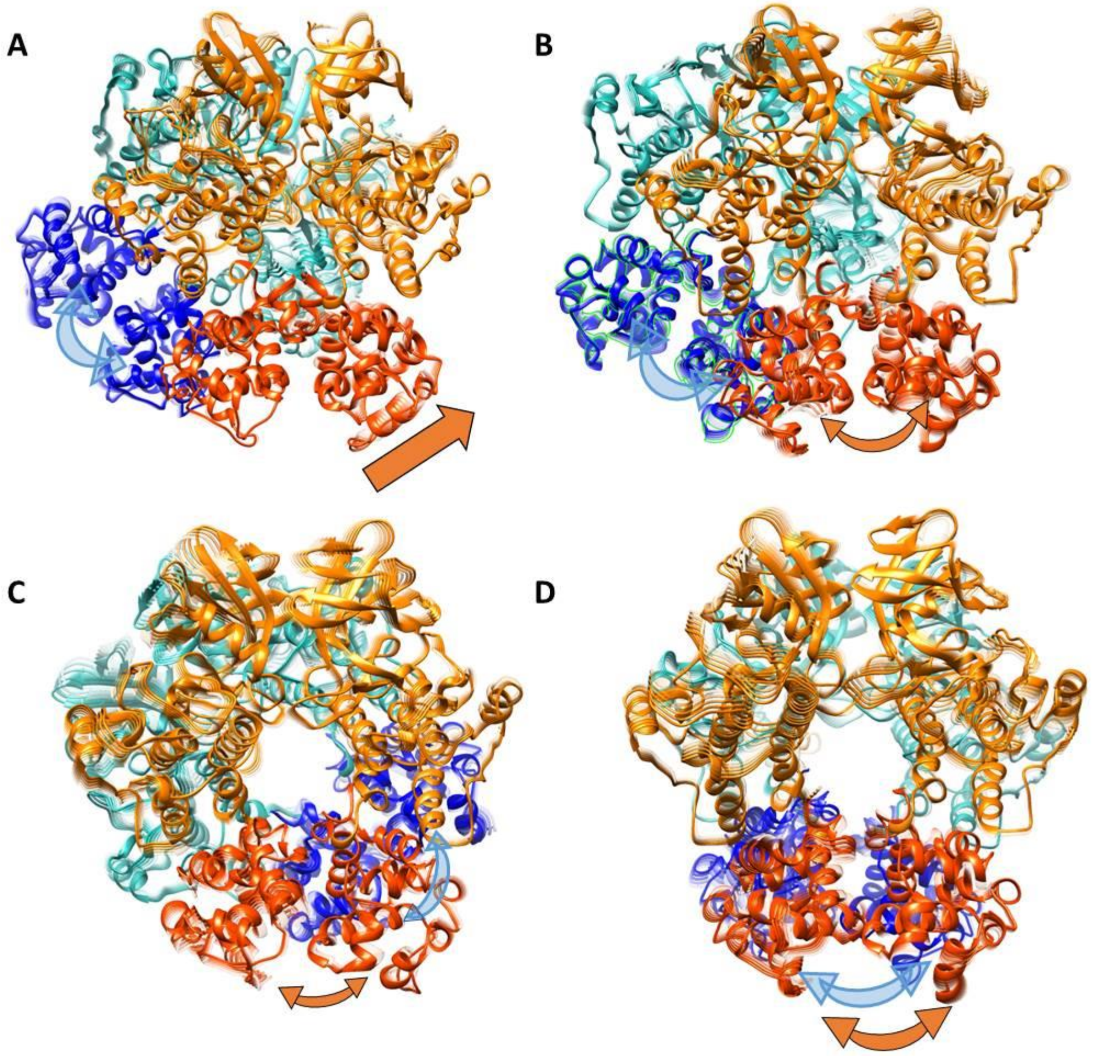

Figure S13. Motion of PC2 PCA obtained from MD simulations of IRE1 heavy atoms of (A) *y*IRE1$_4$ and human IRE1 tetramer models: (B) *h*IRE1$_4$(R), (C) *h*IRE1$_4$(L), and (D) *h*IRE1$_4$(S). The straight arrows indicate IRE1 RNase region breathing expressed in opening of each dimer in the RNase domain while the curved arrows describe tilting motion within each RNase domain in the RNase domain. The kinase domains are shown in orange and light green and the RNase domains in red and blue, respectively. Protein images produced using UCSF Chimera 1.14, https://www.cgl.ucsf.edu/chimera.

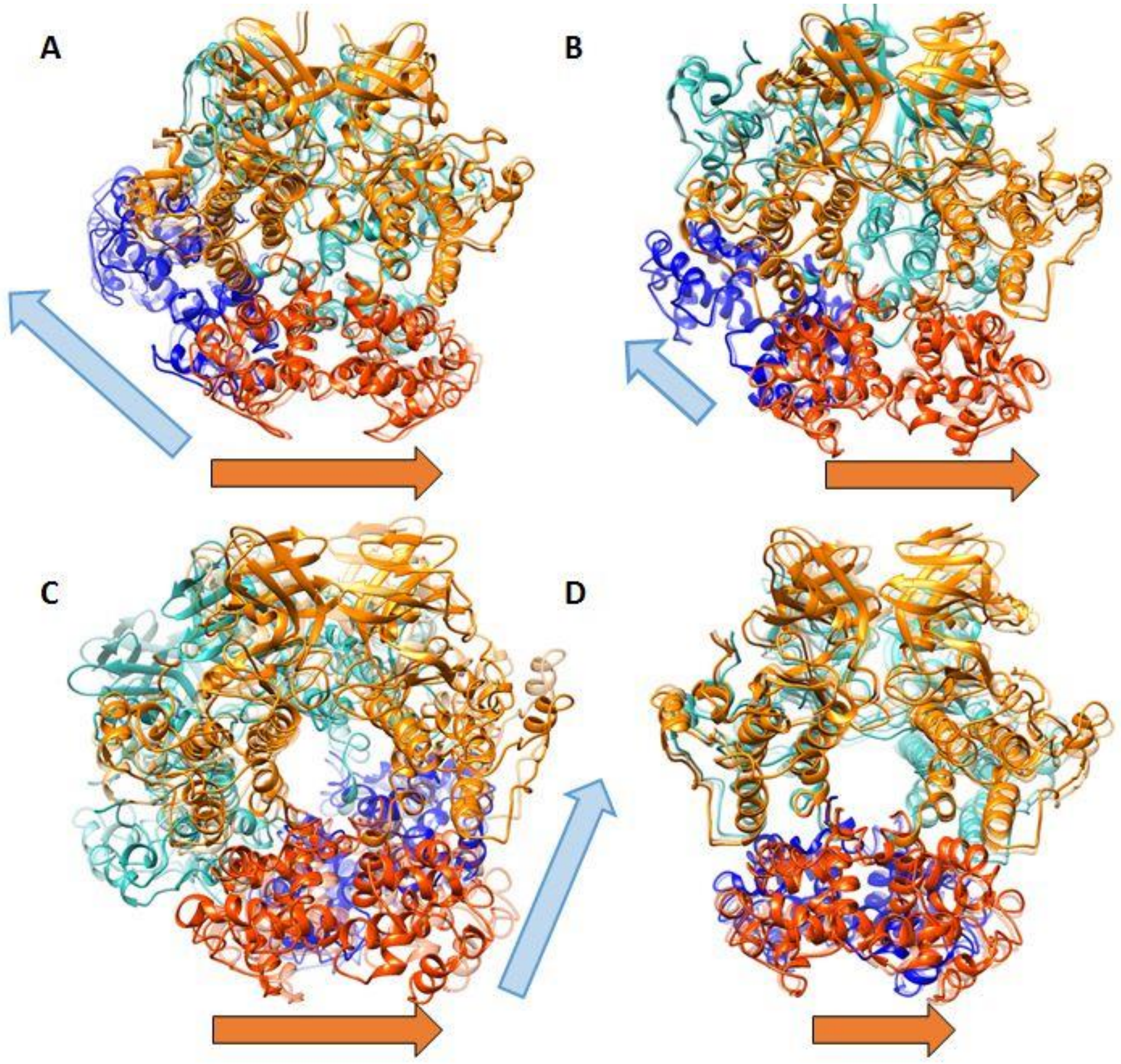

Figure S14. Motion of low mode vibrational sampling of IRE1 heavy atoms of (A) *y*IRE1$_4$ and human IRE1 tetramer models: (B) *h*IRE1$_4$(R), (C) *h*IRE1$_4$(L), and (D) *h*IRE1$_4$(S). The straight arrows indicate IRE1 RNase region breathing expressed in opening of each dimer in the RNase domain. The kinase domains are shown in orange and light green and the RNase domains in red and blue, respectively. Protein images produced using UCSF Chimera 1.14, https://www.cgl.ucsf.edu/chimera.

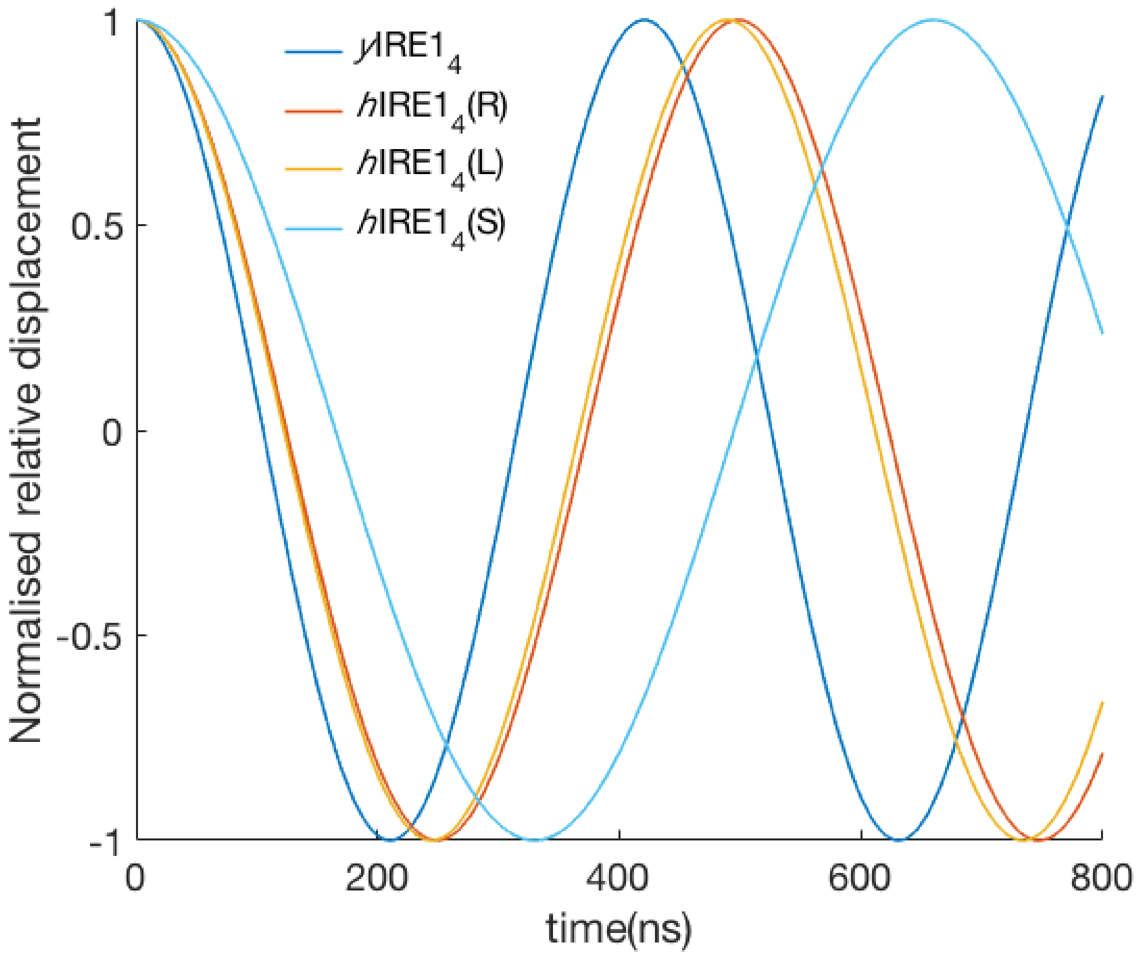

Figure S15. Time frame of the low mode vibrational sampling motions described in Figure S12.

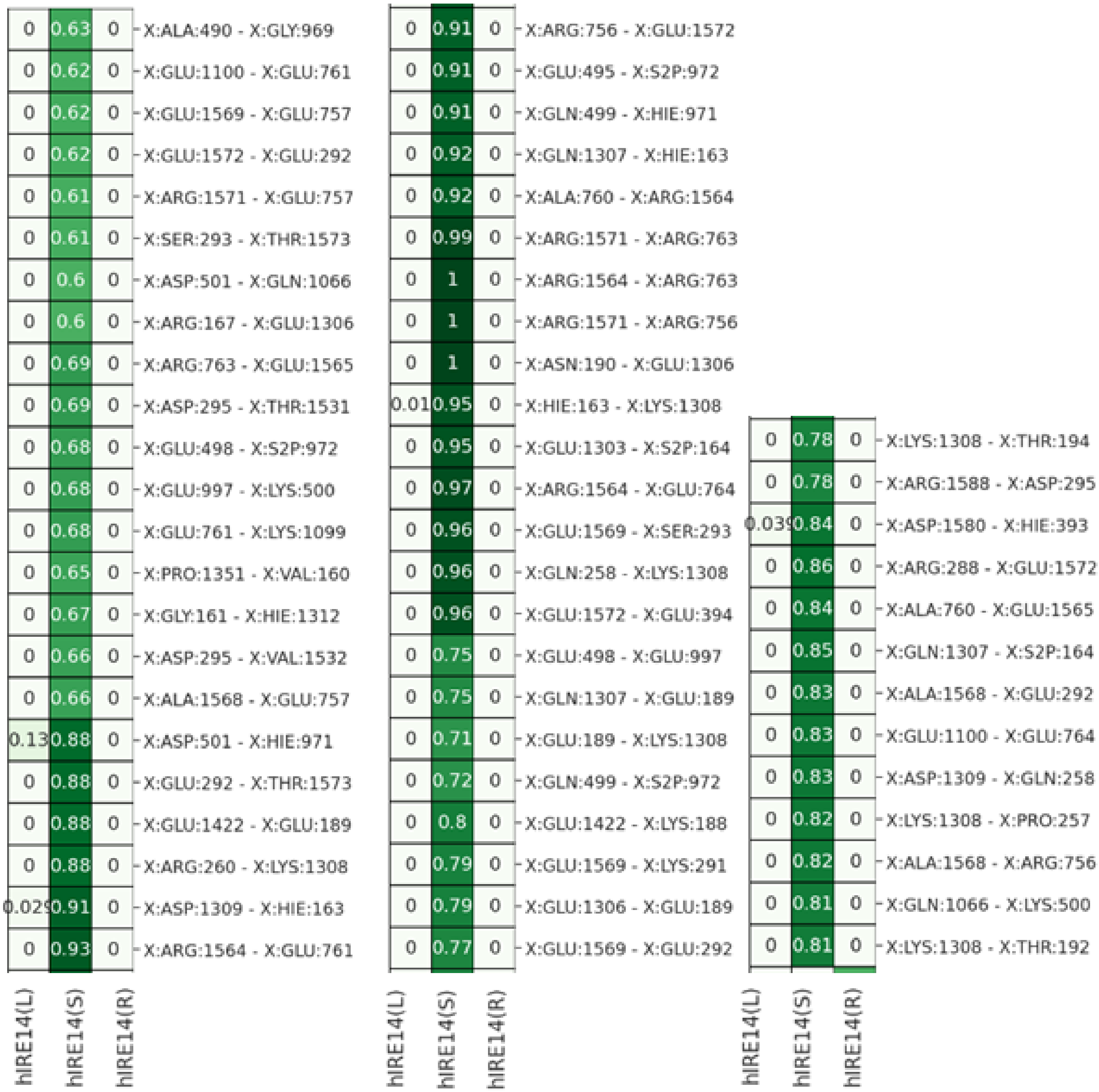

Figure S16. Total contacts between dimer A and dimer B during the MD simulations of *h*IRE1$_4$(S). Normalized frequency of contacts between the three systems is shown.

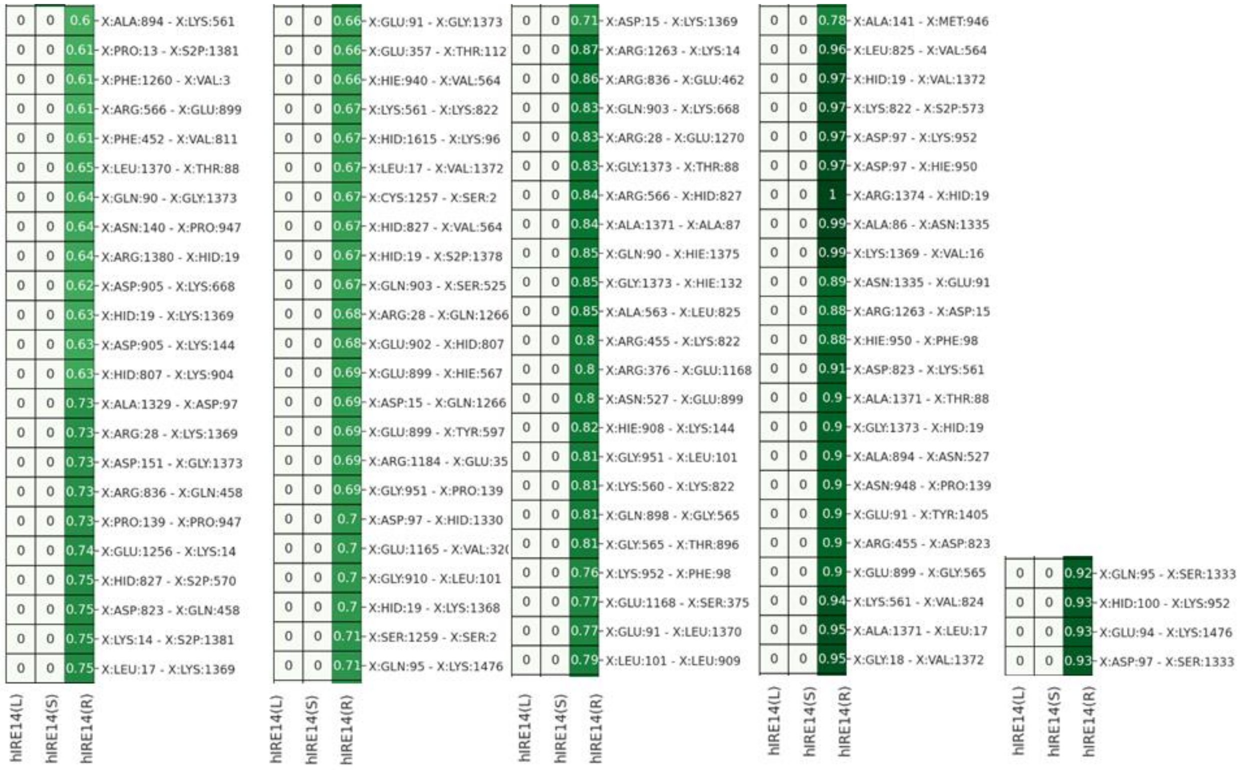

Figure S17. Total contacts between dimer A and dimer B during the MD simulations of *h*IRE1$_4$(R). Normalized frequency contacts between the three systems is shown.

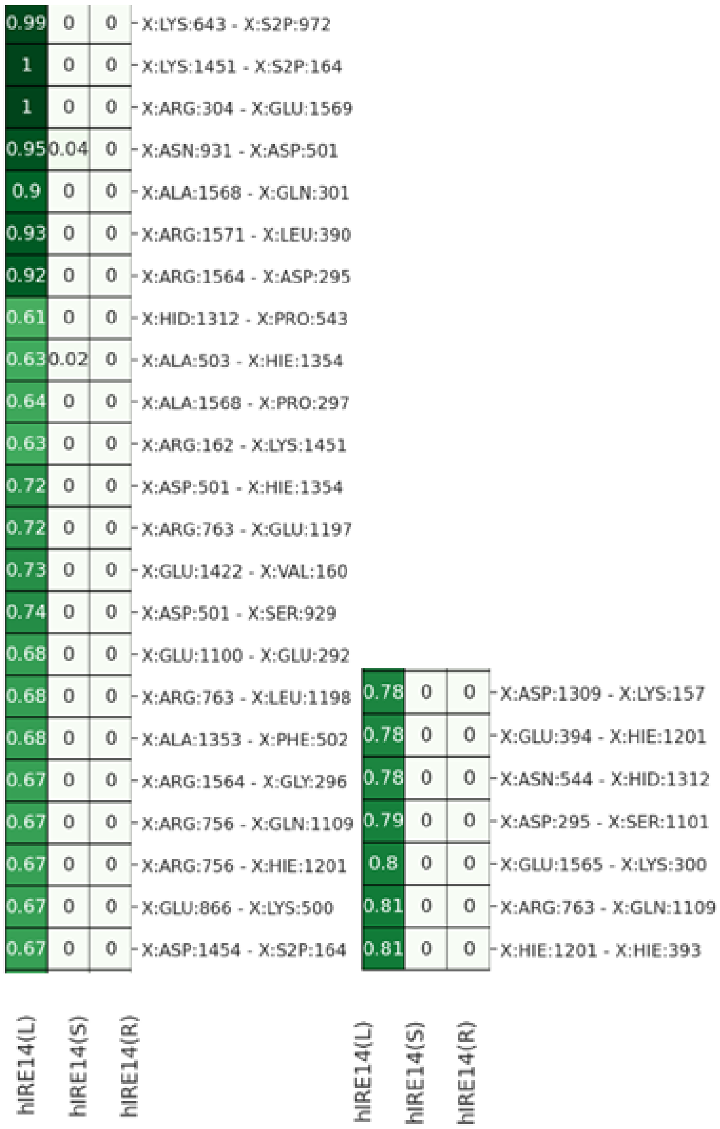

Figure S18. Total contacts between dimer A and dimer B during the MD simulations of *h*IRE1$_4$(L). Normalized frequency contacts between the three systems is shown.

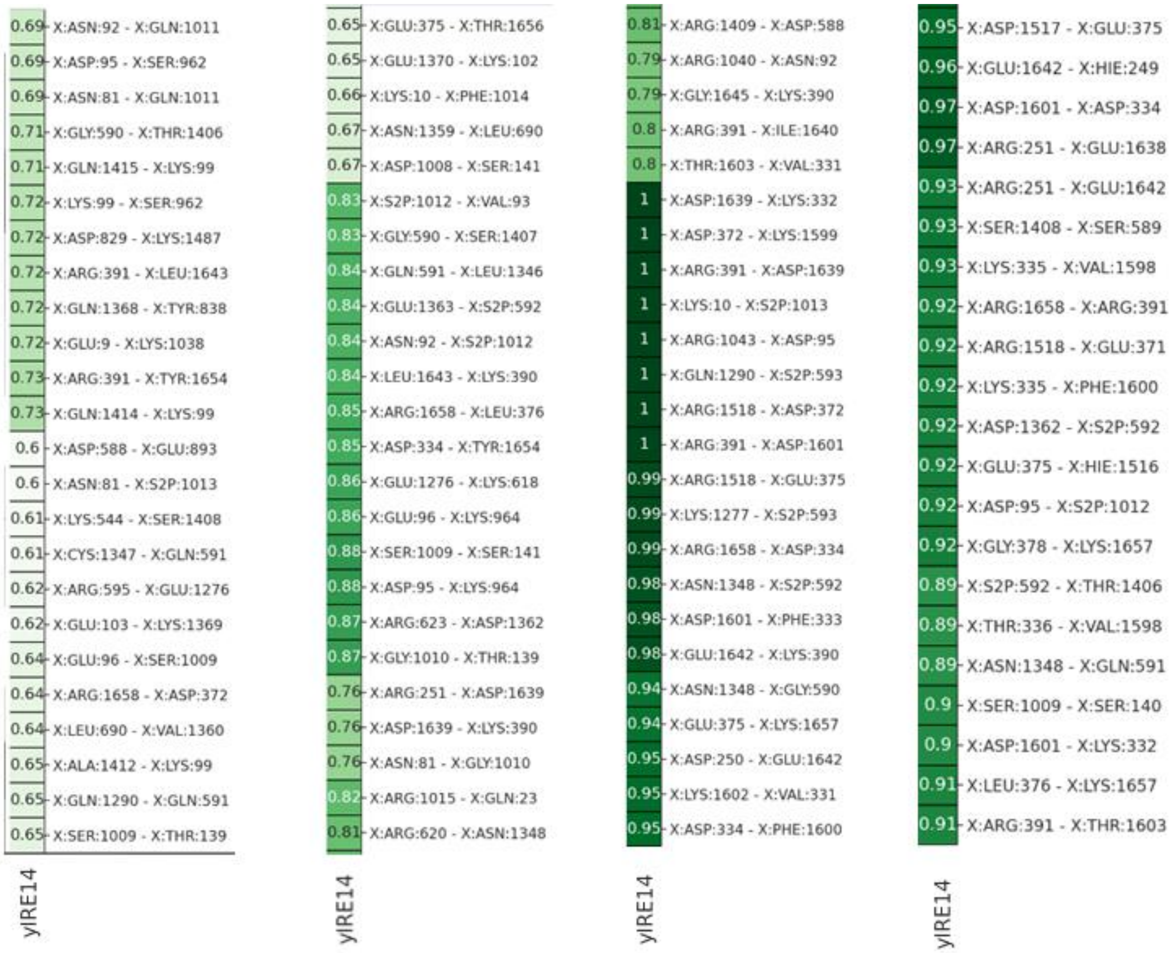

Figure S19. Total contacts between dimer A and dimer B during the MD simulations of *y*IRE1$_4$.
